# CAD-score-LT: versatile contact area-based comparison of macromolecular structures, interfaces, and binding sites

**DOI:** 10.1093/bioinformatics/btag756

**Published:** 2026-10-12

**Authors:** Kliment Olechnovič

**Affiliations:** Institute of Biotechnology, Life Sciences Center, Vilnius University, Vilnius 10257, Lithuania

## Abstract

**Motivation:**

Comparison of macromolecular structural models requires similarity measures that remain informative for complexes, interfaces, and binding sites. Widely used scores emphasize global superposition or distance deviations and are not designed primarily for direct assessment of contact patterns. A fast and flexible contact-area-based alternative is therefore highly relevant.

**Results:**

We present CAD-score-LT, a high-performance implementation of the Contact Area Difference score (CAD-score) for superposition-free comparison of macromolecular structures. CAD-score-LT evaluates tessellation-derived contact, binding-site, and solvent-accessible surface areas at atom, residue, and chain levels, and supports selective scoring of substructures, interfaces, and binding regions. The software also provides sequence-guided preprocessing, chain remapping, symmetry-adjusted ligand scoring, local scores output, and clustering. Benchmarking against previous CAD-score implementations on CASP16 oligomer model pairs showed near-identical scores but more than one order of magnitude lower running times, with speedups reaching up to 100-fold for whole-structure scoring and 200-fold for inter-chain interface scoring. Case studies in supplementary material demonstrate utility for large-scale model clustering, analysis of protein–nucleic acid interfaces, and evaluation of protein–ligand docking models.

**Availability and implementation:**

A repository with the CAD-score-LT software and the reproducible benchmarking and example workflows is available at https://github.com/kliment-olechnovic/cadscore-lt-supplementary-info and archived at Zenodo https://doi.org/10.5281/zenodo.21983528. The latest CAD-score-LT software is openly available under MIT license as a standalone C++ application at https://www.voronota.com/expansion_lt_cadscore/, and as a Python library at https://pypi.org/project/cadscorelt/.

## 1 Introduction

Accurate comparison of macromolecular structures with identical (or consistently numbered) sequences is essential for the development, benchmarking, and self-assessment of structure prediction and analysis methods. Although prediction of monomeric protein structures improved markedly with AlphaFold2 (Jumper *et al.* 2021), prediction of protein–protein, protein–nucleic acid, and protein–ligand complexes remains substantially more difficult, as illustrated by recent CASP and CAPRI assessments and large-scale benchmarking studies (Škrinjar *et al.* 2026, Zhang *et al.* 2026, Lensink *et al.* 2026). This problem requires similarity measures that remain informative for intermolecular contacts and functional regions.

The most widely used scores, such as TM-score (Zhang and Skolnick 2004) and lDDT (Mariani *et al.* 2013), have broad utility, but they do not directly quantify agreement of contact areas. Contact area difference (CAD) was introduced as a robust alternative in 1997 (Abagyan and Totrov 1997) and was later reformulated using Voronoi tessellation-derived contact areas to yield CAD-score (Olechnovič *et al.* 2013) for proteins and nucleic acids (Olechnovič and Venclovas 2014). CAD-score has since been used in CASP and CAMEO evaluation workflows (Kryshtafovych *et al.* 2014, Robin *et al.* 2021, Studer *et al.* 2026), shown sensitivity to important features of protein models (Olechnovič *et al.* 2019), and supported downstream applications such as complex clustering (Dapkūnas *et al.* 2024) and docking model analysis (Olechnovič *et al.* 2026).

Previous CAD-score implementations, however, were relatively slow, largely limited to residue–residue contacts, inconvenient for selective scoring of interfaces or other subsets, and difficult to integrate into modern analysis pipelines. Here we present CAD-score-LT, a new implementation that addresses these limitations. In addition to efficient global scoring, CAD-score-LT generalizes the CAD framework to multiple tessellation-derived area types and multiple levels of structural detail.

## 2 Materials and methods

### 2.1 Calculation of CAD-score

CAD-score can be defined for any set of structure-derived objects with matched identifiers and associated areas. Given a reference structure *T* and a model *M*, let *G* be the set of all objects *i* with non-zero area $T_{\left(i\right)}$ in the reference. For each i∈G, the corresponding area $M_{\left(i\right)}$ is obtained from the model; if the object is absent or has no corresponding area, $M_{\left(i\right)}$ is set to zero. CAD-score is then


(1) $$\text{CAD–score}(G)=1-\frac{\sum_{i\in G}\text{min}(|T_{\left(i\right)}-M_{\left(i\right)}|,T_{\left(i\right)})}{\sum_{i\in G}T_{\left(i\right)}}.$$

In CAD-score-LT this formulation is applied to three tessellation-derived area classes: contact areas, binding-site areas accumulated in the presence of binding partners, and solvent-accessible surface (SAS) areas for unbound regions. All areas are derived from atom-level contact calculations performed with Voronota-LT (Olechnovič and Grudinin 2025) and can be aggregated to atom, residue, or chain level, yielding nine scoring modes (Fig. 1). The set *G* can be restricted with a simple selection language, allowing scoring of complete structures, specific substructures, interfaces, or binding sites. When multiple interfaces are selected, their area terms are included together in *G* and a single CAD-score is calculated over the complete set, rather than by averaging per-interface scores. In addition to CAD-score, CAD-score-LT reports true-positive, false-positive, and false-negative areas, from which an area-based F1 score can be derived.

**Figure 1:**
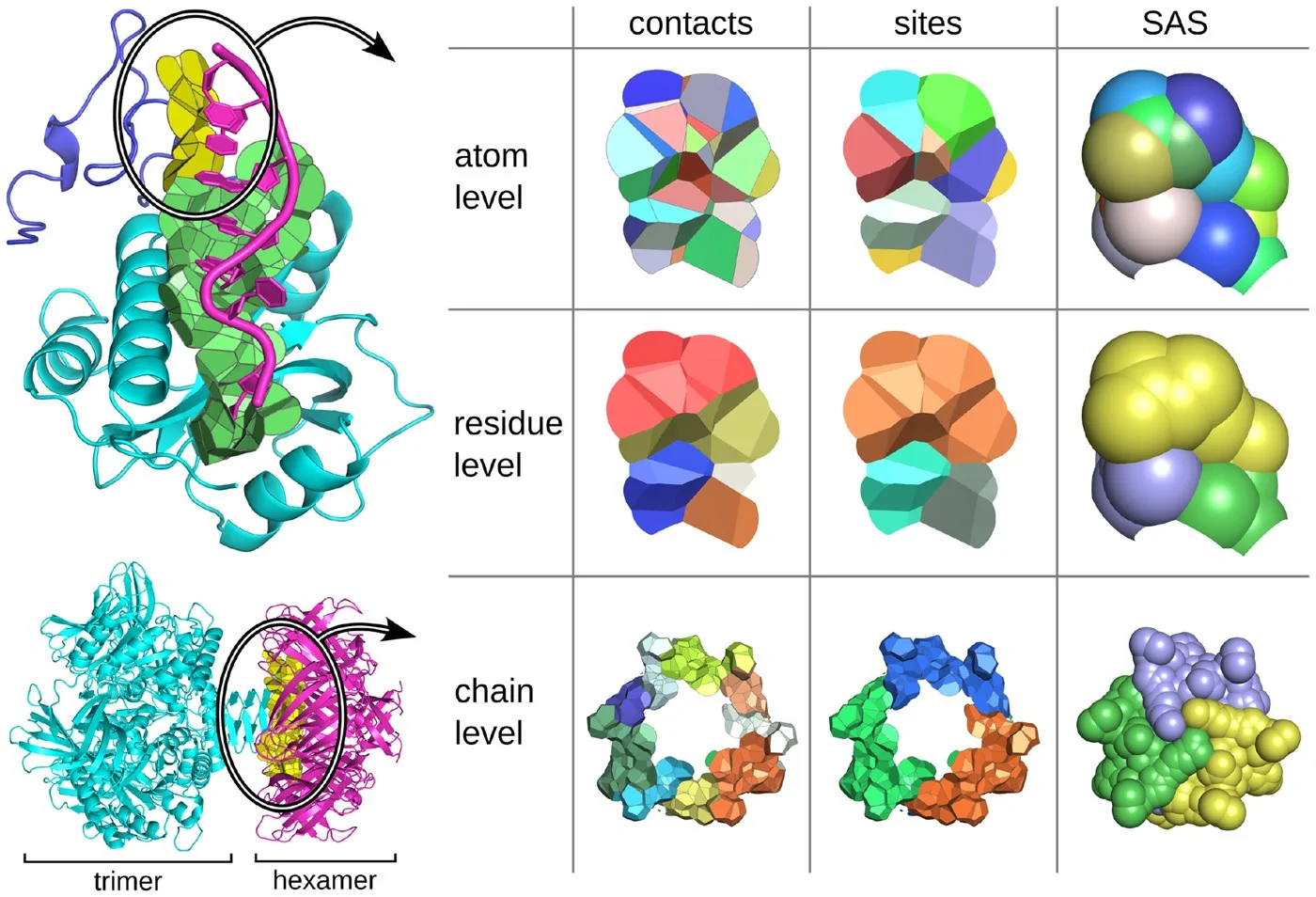
Types of areas that can be compared with CAD-score-LT. The atom- and residue-level examples are based on the sub-interface between the N-terminus of the YTH-domain protein Mmi1 and the RNA chain from PDB entry 6FPQ (Stowell *et al.* 2018)—the site and SAS examples are given for the protein N-terminus side. The chain-level examples are based on the trimer-with-hexamer interface in the Haloferax tailed virus 1-related complex from PDB entry 8QPQ (Zhang *et al.* 2025)—the site and SAS examples are given for the trimer side.

### 2.2 Preparation of input structures

Reliable CAD-score calculation requires consistent identifiers between reference and model structures. CAD-score-LT therefore supports automatic assignment of residue numbering and chain identifiers from reference sequences, using Needleman–Wunsch sequence alignment (Needleman and Wunsch 1970) and optional reference stoichiometry.

Atom-level ligand assessment poses additional challenges. CAD-score-LT includes companion scripts that assign deterministic canonical ligand atom names (using RDKit canonical atom ranking) and that generate equivalence classes for symmetric ligand atoms. These equivalence classes enable symmetry-adjusted atom-level scoring by summing areas over equivalent atoms.

### 2.3 Handling mismatched identifiers

By default, residue names are not treated as part of residue or atom identifiers, which allows comparison of structures with conserved numbering but different residue identities. Contact areas are still calculated from all atoms present in each structure, so differences between side chains affect the resulting areas and scores rather than preventing the corresponding residues from being compared. The software can also report the fractions of matched atom, residue, and chain identifiers.

For multimeric complexes, inconsistent chain naming can substantially decrease similarity scores. CAD-score-LT therefore supports automatic chain remapping to identify mappings with higher CAD-score. Remapping is exhaustive when the number of candidate assignments is below a configurable threshold (200 by default). For larger search spaces, a fast greedy procedure constructs the mapping one chain at a time by maximizing contact-area overlap. The greedy procedure is not guaranteed to identify the globally highest possible CAD-score.

### 2.4 Additional functionality

Additional functionality includes clustering with a modified Taylor–Butina algorithm (Dapkūnas *et al.* 2024), local score output at atom, residue, and chain levels, export of scores to PDB/mmCIF B-factors, tessellation graphics for PyMOL or ChimeraX, SVG contact map plots, OpenMP parallelization, and Python bindings generated with SWIG. The software is implemented in self-contained C++ and does not depend on legacy CAD-score code or shell-script wrappers.

## 3 Benchmarking and case studies

Benchmarking against two previous CAD-score implementations on 12 309 randomly sampled CASP16 oligomer model pairs showed that CAD-score-LT reproduces previous whole-structure and inter-chain interface scores with very high fidelity while being consistently much faster (Section 2, available as supplementary data at *Bioinformatics* online). For whole-structure residue-level scoring, agreement with CAD-score-2012 and CAD-score-2021 reached Pearson correlations of 0.998 and >0.999, respectively; for inter-chain interface scoring the corresponding correlations were 0.998 and >0.999. Mean absolute score differences remained small (0.010 and 0.00055 for whole-structure scoring; 0.014 and 0.00057 for inter-chain scoring). In contrast, running times differed by more than an order of magnitude: CAD-score-LT was up to about 100-fold faster for whole-structure scoring, up to about 200-fold faster than CAD-score-2012 and 30-fold faster than CAD-score-2021 for inter-chain interface scoring, and remained faster even when compared with a modified CAD-score-2021 version using the same Voronota-LT geometric engine. Using the same CASP16 dataset, we also benchmarked automatic chain remapping and compared inter-chain CAD-scores with established complex and interface measures (Sections 3 and 4, available as supplementary data at *Bioinformatics* online). CAD-score correlated strongly with ilDDT (r=0.98), ICS (r=0.95), QS-global (r=0.92), and DockQ-wave-full (r=0.89), while its correlation with TM-score was lower (r=0.68). The high correlation with ilDDT on this dataset likely reflects that both scores are sensitive to local interface geometry, whereas the lower correlation with TM-score is consistent with TM-score’s superposition-based emphasis on overall structural similarity rather than direct contact agreement. Despite this high overall correlation, the two scores can differ substantially for individual model pairs (Fig. 7a, available as supplementary data at *Bioinformatics* online), and therefore are not numerically interchangeable. ilDDT evaluates local distance agreement, whereas CAD-score directly compares tessellation-derived contact areas and additionally supports binding-site analysis and scoring at atom, residue, and chain levels. Comparison with OpenStructure (Studer *et al.* 2026) further showed similar score-level effects of chain remapping for the two independently implemented procedures.

Four case studies illustrate the scope of the method. First, all-against-all inter-chain interface comparison of 8040 MassiveFold (Raouraoua *et al.* 2026) models for CASP16 target H1202 showed that preprocessing and chain remapping were important for recovering comparable interfaces and enabled rapid clustering of the ensemble into major structural groups (Section 5, available as supplementary data at *Bioinformatics* online). Second, analysis of 122 very large models of CASP17 target H2424 (stoichiometry A_3_B_3_C_6_D_18_E_3_F_3_) demonstrated practical comparison and clustering of complexes with complex stoichiometry and high-order repeated-chain components (Section 6, available as supplementary data at *Bioinformatics* online). Third, analysis of CASP16 target M1239 showed that CAD-score-LT can separately assess protein–protein and protein–DNA interface quality, quantify binding-site accuracy independently from detailed contact accuracy, and provide informative local residue-level views of predicted interfaces (Section 7, available as supplementary data at *Bioinformatics* online). Fourth, evaluation of CASF-2016 (Su *et al.* 2019) protein–ligand docking decoys showed that atom-level and binding-site CAD-scores complement RMSD-based assessment and that symmetry-adjusted ligand scoring substantially improves interpretation when equivalent ligand atoms are present (Section 8, available as supplementary data at *Bioinformatics* online).

## 4 Conclusions

CAD-score-LT extends CAD-score from a residue-contact similarity measure into a general framework for fast, selective, superposition-free comparison of structures, interfaces, and binding sites. The software combines broad scoring flexibility with practical features needed for contemporary analysis of structural models of complexes, including preprocessing, chain remapping, ligand symmetry handling, local scoring outputs, and clustering. Benchmarking indicates that these gains are achieved while substantially reducing running time, reproducing the behavior of earlier CAD-score implementations, and remaining correlated with several established complex and interface similarity measures. CAD-score-LT should therefore be useful both as a high-performance replacement for previous CAD-score workflows and as a general-purpose tool for analysis of large sets of macromolecular conformations.

## Supplementary Material

btag756_Supplementary_Data

## Data Availability

A repository with the CAD-score-LT software and the reproducible benchmarking and example workflows is available at https://github.com/kliment-olechnovic/cadscore-lt-supplementary-info, archived at Zenodo https://doi.org/10.5281/zenodo.21983528, and summarized in Section 1, available as supplementary data at *Bioinformatics* online. The latest CAD-score-LT software is openly available under MIT license at https://www.voronota.com/expansion_lt_cadscore/.
