# Supplementary material for: CAD-score-LT: versatile contact area-based comparison of macromolecular structures, interfaces, and binding sites
Source: Bioinformatics. 2026 Oct 12;42(10):btag756. doi: 10.1093/bioinformatics/btag756 (PMC13686761; doi:10.1093/bioinformatics/btag756)
Supplement: Supplementary file 1 [file btag756_supplementary_data.pdf]

Supplementary information for

CAD-score-LT: versatile contact area-based comparison of macromolecular structures, interfaces, and binding sites

Kliment Olechnovič,[1,*]

[1]Institute of Biotechnology, Life Sciences Center, Vilnius University, 10257 Vilnius, Lithuania

[*]To whom correspondence should be addressed. Email: kliment.olechnovic@bti.vu.lt

S1 Overview of the workflows used for benchmarking and example applications

Sections S2–S8 present the benchmarking and example applications of CAD-score-LT. All workflows used to perform the relevant computations and generate the summary results and figures are provided in reproducible form in the supplementary GitHub repository at https://github.com/kliment-olechnovic/cadscore-lt-supplementary-info. The repository is archived at version 1.0.0 on Zenodo with the DOI https://doi.org/10.5281/zenodo.21983528.

The correspondence between the sections and the repository subdirectories is as follows:

- /supplementary_workflows
  - /comparison_with_different_scores — sections S2, S3, S4;
  - /massivefold_clustering_example — section S5;
  - /large_complex_clustering_example — section S6;
  - /mixed_complex_analysis — section S7;
  - /protein_ligand_docking_analysis — section S8;

The workflow subdirectories contain the scripts used for the analyses, together with the resulting tables and plots.

The versions of the software packages used in the workflows to compute the scores are as follows:

- CAD-score-LT version 0.9.196 from https://github.com/kliment-olechnovic/voronota/releases/download/v1.29.4816/cadscore-lt_v0.9.196.tar.gz
- "Extra data-preparation utilities for CAD-score-LT" version 0.9.1 from https://github.com/kliment-olechnovic/cadscorelt-extra-data-utilities/archive/refs/tags/v0.9.1.tar.gz
- "old CAD-score" version 1.1662 from https://github.com/kliment-olechnovic/old_cadscore/releases/download/1.1662/cadscore_1.1662.tar.gz
- Voronota version 1.29.4816 from https://github.com/kliment-olechnovic/voronota/releases/download/v1.29.4816/voronota_1.29.4816.tar.gz
- OpenStructure package version 2.11.1 from https://openstructure.org/

The source code of the CAD-score-LT version and the extra data-preparation utilities used in these workflows is also included in the supplementary repository in the /software_source_codes subdirectory. This provides a permanently stored, frozen copy of the software benchmarked for this publication.

The main access point for all future software releases of CAD-score-LT is https://www.voronota.com/expansion_lt_cadscore/index.html.

S2 Benchmarking CAD-score-LT against previous CAD-score implementations

We compared CAD-score-LT with two previous implementations of CAD-score.

- **CAD-score-2012**: the implementation that computes residue–residue contact areas by summing atom-level contact areas derived from Voronoi tessellation of atomic balls, with atom contact areas estimated by sphere subdivision over tessellation neighbors. The software is hosted at https://github.com/kliment-olechnovic/old_cadscore.

- **CAD-score-2021**: the implementation in Voronota-JS (https://www.voronota.com/expansion_js/index.html) that computes residue–residue contact areas by summing atom-level areas corresponding directly to Voronoi cell faces constrained inside the solvent-accessible surface. This contact construction mode is used in the PPI3D server and in the FTDMP framework.

Both earlier implementations can compare inter-chain interfaces. However, CAD-score-2012 always requires construction of the full Voronoi tessellation, whereas CAD-score-2021 attempts to construct only the minimal sub-tessellation required for a given inter-chain interface.

Benchmarking used data from CASP16. We downloaded all collections of oligomeric protein and RNA models from https://predictioncenter.org/download_area/CASP16/predictions/oligo/. From each collection, 100 random structure pairs were selected. Within each pair, one structure was designated as the target and the other as the model. Because the older implementations are slow for very large inputs, pairs with more than 100,000 atoms in total were excluded. The final benchmark set contained 12,309 pairs.

For each pair we evaluated two regimes: whole-structure residue–residue contacts and inter-chain residue–residue contacts. The resulting score comparisons are shown in Figs. S1 and S2. Agreement between implementations was very high.

For whole-structure scores:

- CAD-score-LT versus CAD-score-2012: Pearson correlation coefficient 0.998; mean absolute score difference 0.010.

- CAD-score-LT versus CAD-score-2021: Pearson correlation coefficient > 0.999; mean absolute score difference 0.00055.

For inter-chain interface scores:

- CAD-score-LT versus CAD-score-2012: Pearson correlation coefficient 0.998; mean absolute score difference 0.014.

- CAD-score-LT versus CAD-score-2021: Pearson correlation coefficient > 0.999; mean absolute score difference 0.00057.

Although the score differences were small, running times differed markedly (Fig. S3). CAD-score-LT was consistently the fastest implementation, typically by more than one order of magnitude. For whole-structure assessment, speedups reached about 100-fold relative to CAD-score-2012 or CAD-score-2021. For inter-chain interface assessment,

speedups reached about 200-fold relative to CAD-score-2012 and about 30-fold relative to CAD-score-2021.

To separate gains due to the geometric engine from gains due to the overall implementation, we also replaced the contact-area calculation routine in CAD-score-2021 with the Voronota-LT routine used in CAD-score-LT. This modified version, denoted CAD-score-2021-LT-mod, was still slower than CAD-score-LT (Fig. S4), especially for inter-chain interface assessment, where the speedup reached about sevenfold. Thus, the performance improvement of CAD-score-LT does not arise solely from the use of Voronota-LT.

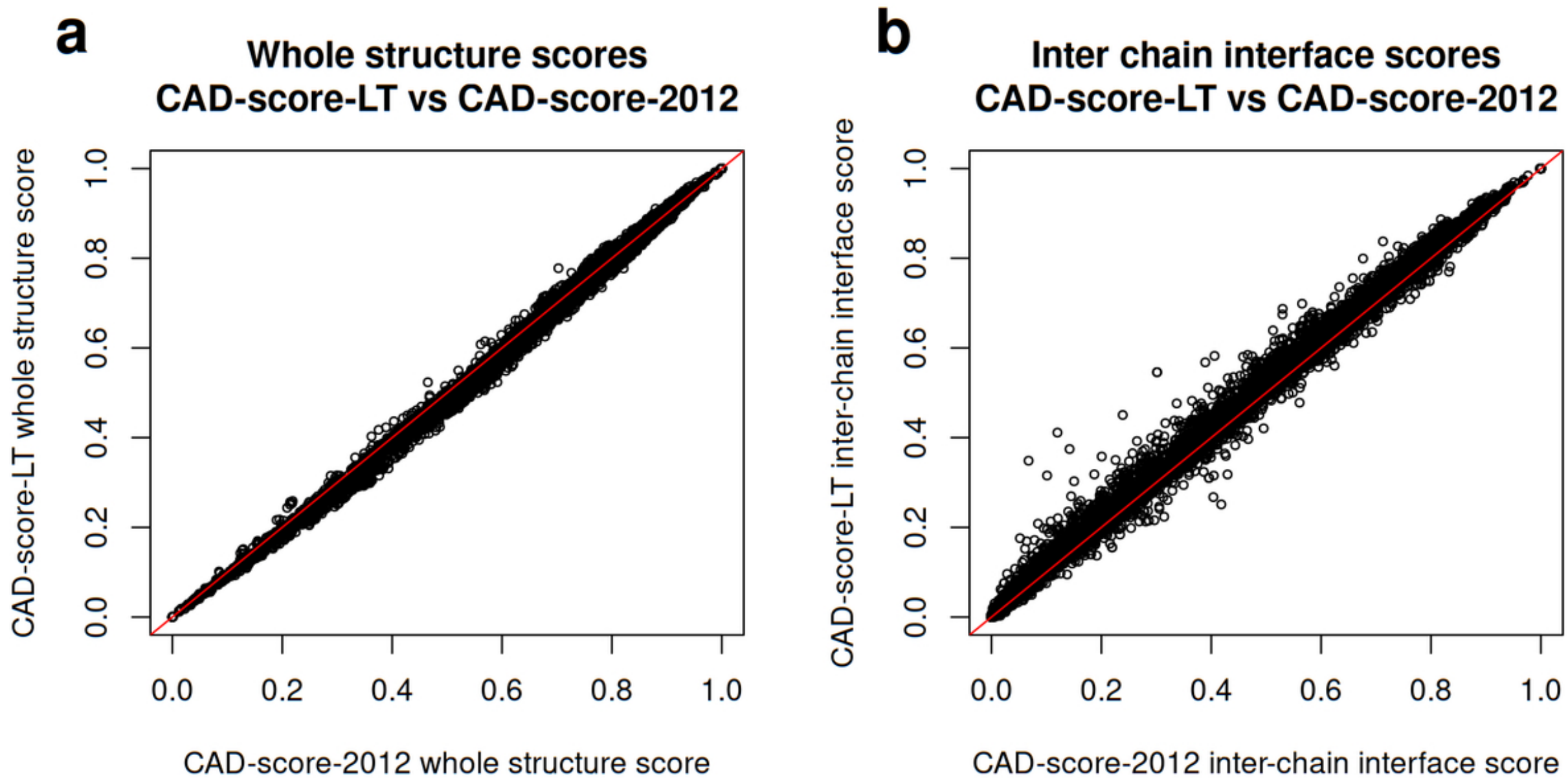

Fig. S1. Correlation between whole-structure (a) and inter-chain interface (b) scores produced by CAD-score-LT and CAD-score-2012 for CASP16 oligomeric predictions.

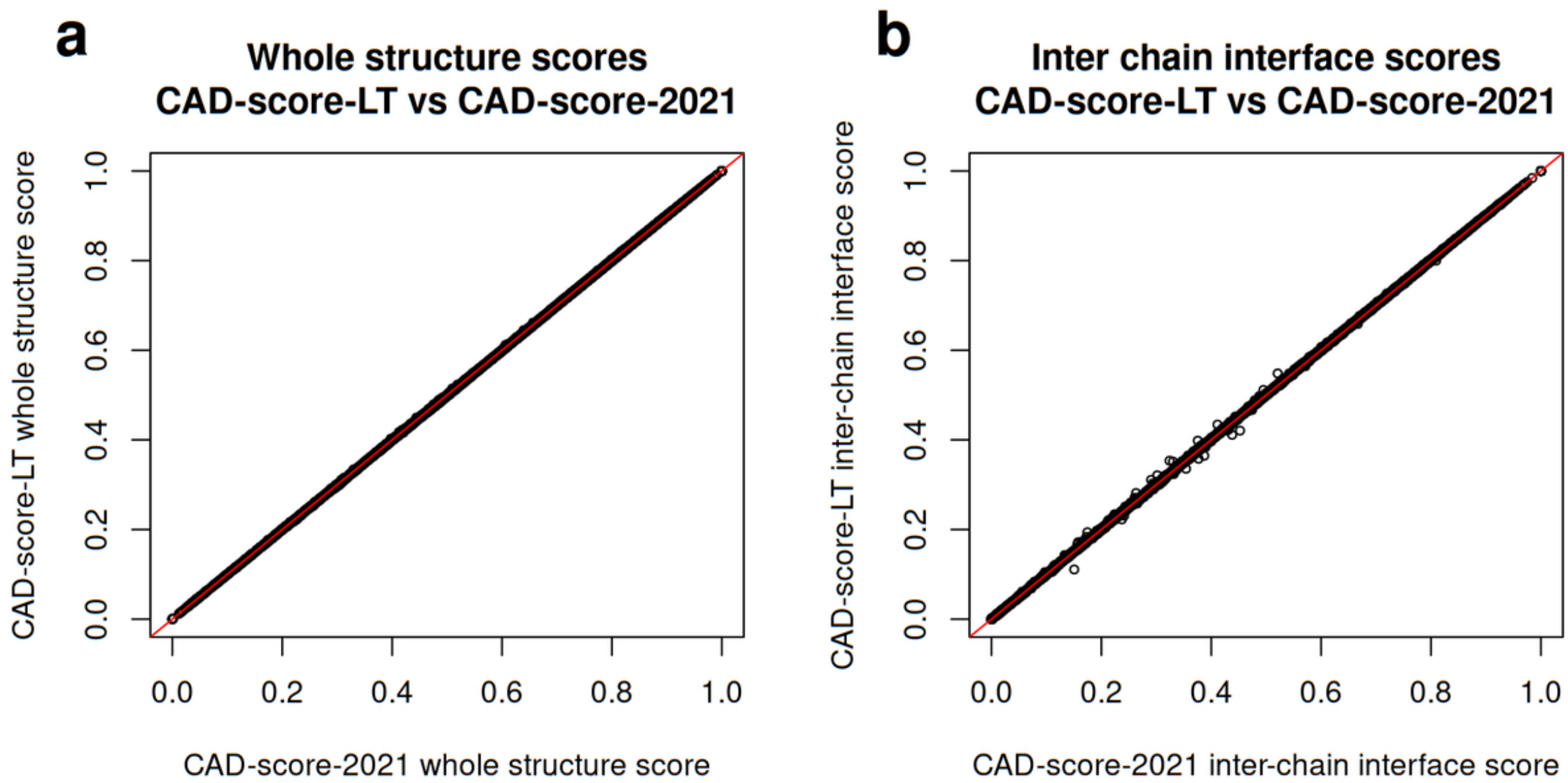

Fig. S2. Correlation between whole-structure (a) and inter-chain interface (b) scores produced by CAD-score-LT and CAD-score-2021 for CASP16 oligomeric predictions.

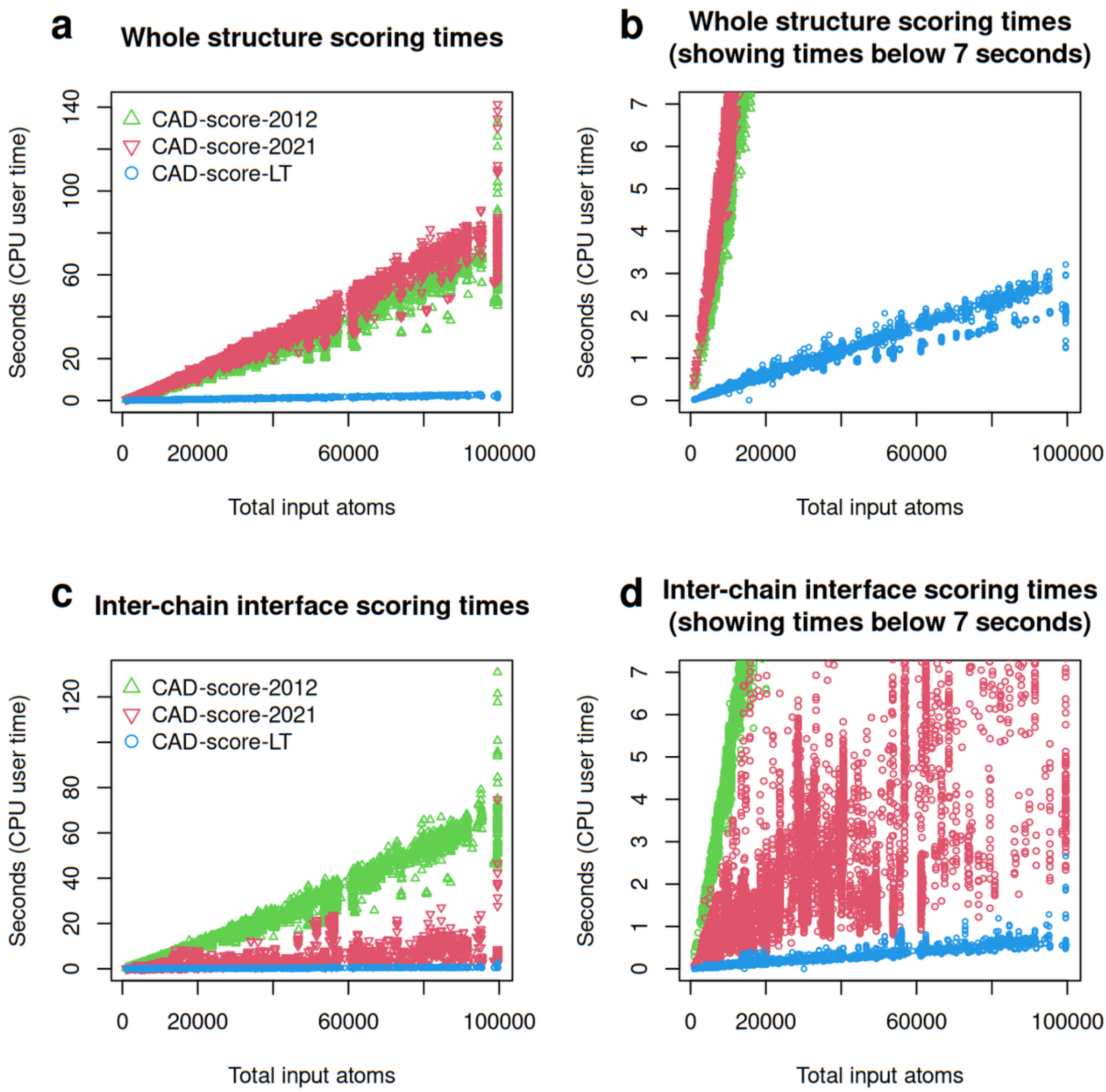

Fig. S3. Running times (CPU user times) of different CAD-score implementations when comparing whole structures (a, b) and inter-chain interfaces (c, d) of CASP16 oligomeric predictions.

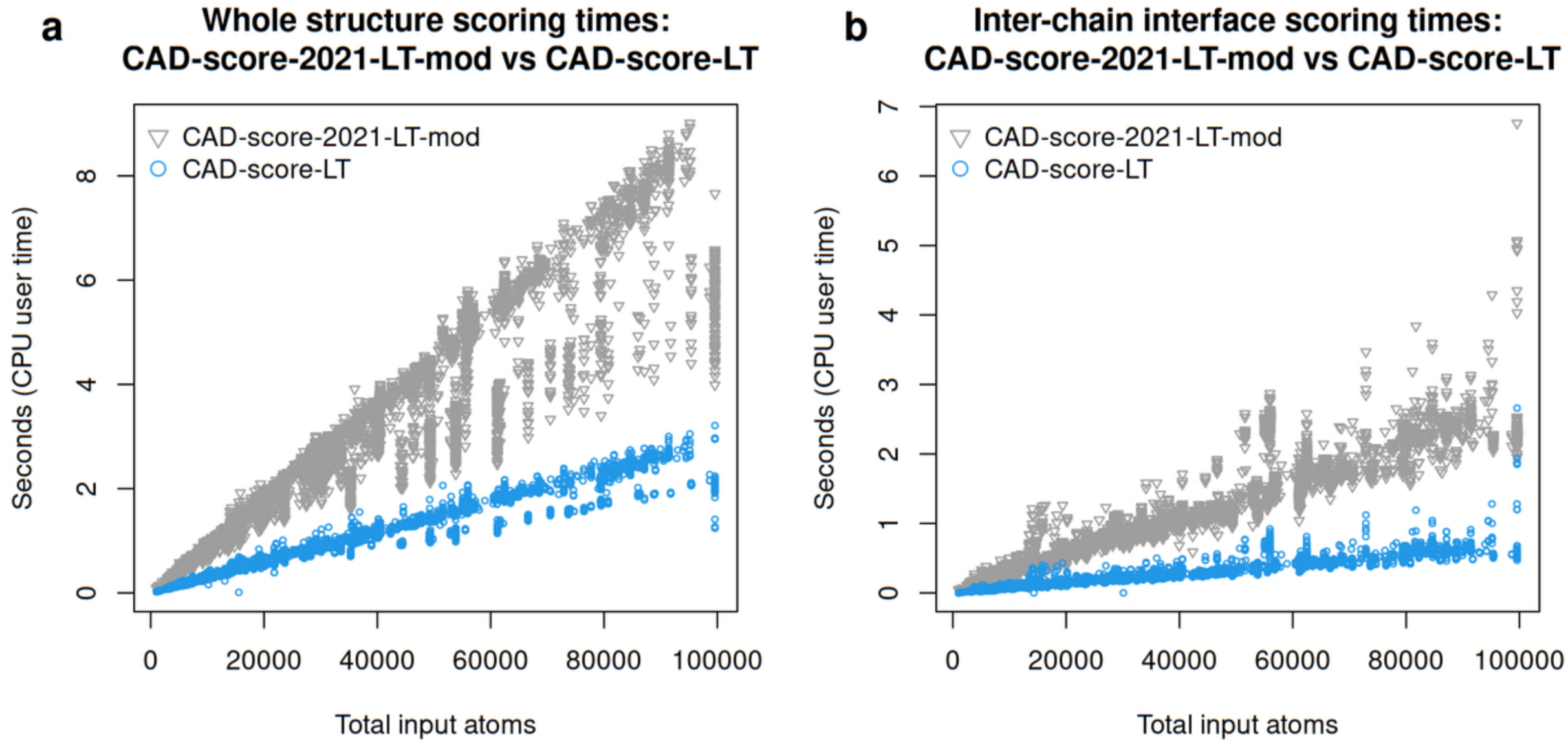

Fig. S4. Running times (CPU user times) of CAD-score-LT and a modified CAD-score-2021 implementation using Voronota-LT instead of Voronota for tessellation-derived area computation, recorded for whole-structure (a) and inter-chain interface (b) comparisons of CASP16 oligomeric predictions.

S3 Benchmarking CAD-score-LT integrated chain remapping

Using the same input structural data as in section S2, we used CAD-score-LT to calculate residue–residue contact CAD-scores under the following conditions:

1. preprocessing with automatic residue renumbering and chain naming based on reference sequences, which was enabled in all cases;

2. automatic chain remapping for each pairwise comparison, which was evaluated both enabled and disabled.

Automatic chain remapping does not necessarily guarantee the globally highest possible CAD-score. When the number of candidate chain remappings to consider is smaller than the value specified by the `--max-exhaustive-remappings` parameter (200 by default), CAD-score-LT evaluates the remappings exhaustively. For larger search spaces, it uses a greedy procedure that constructs the mapping one chain at a time, at each step selecting the assignment that maximizes the overlap of contact areas. Thus, the analyses below characterize the practical behavior of the implemented chain-remapping procedure rather than assume that it always identifies the globally optimal remapping.

Enabling automatic chain remapping often resulted in higher, and never lower, CAD-score values at both the whole-structure level (Fig. S5a) and the inter-chain interface level (Fig. S5b).

Importantly, chain remapping did not dramatically increase the running times. The slowdown was more pronounced for whole-structure comparisons (Fig. S6a) and substantially smaller for inter-chain interface comparisons (Fig. S6b).

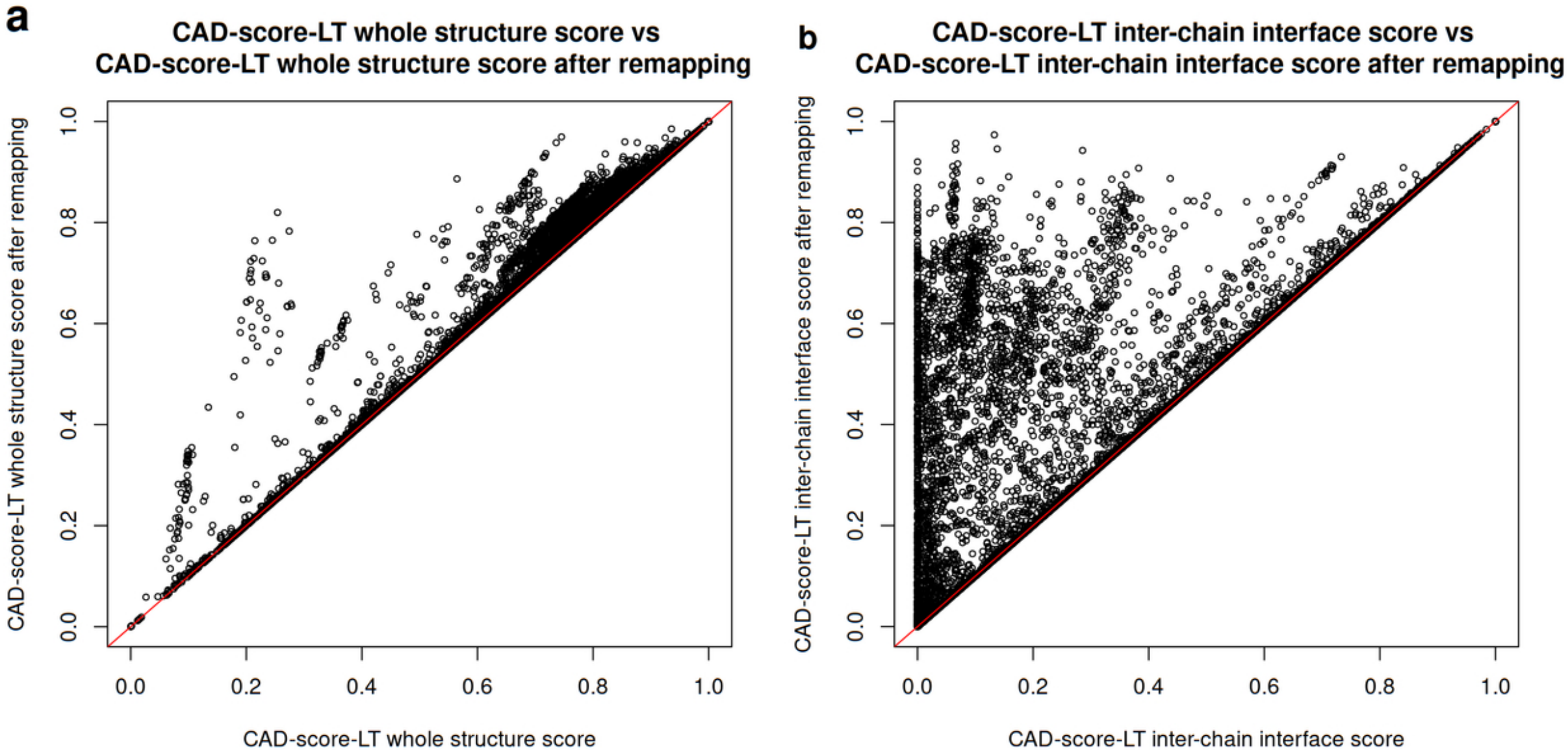

Fig. S5. CAD-score-LT scores with and without automatic chain remapping, recorded for whole-structure (a) and inter-chain interface (b) pairwise comparisons.

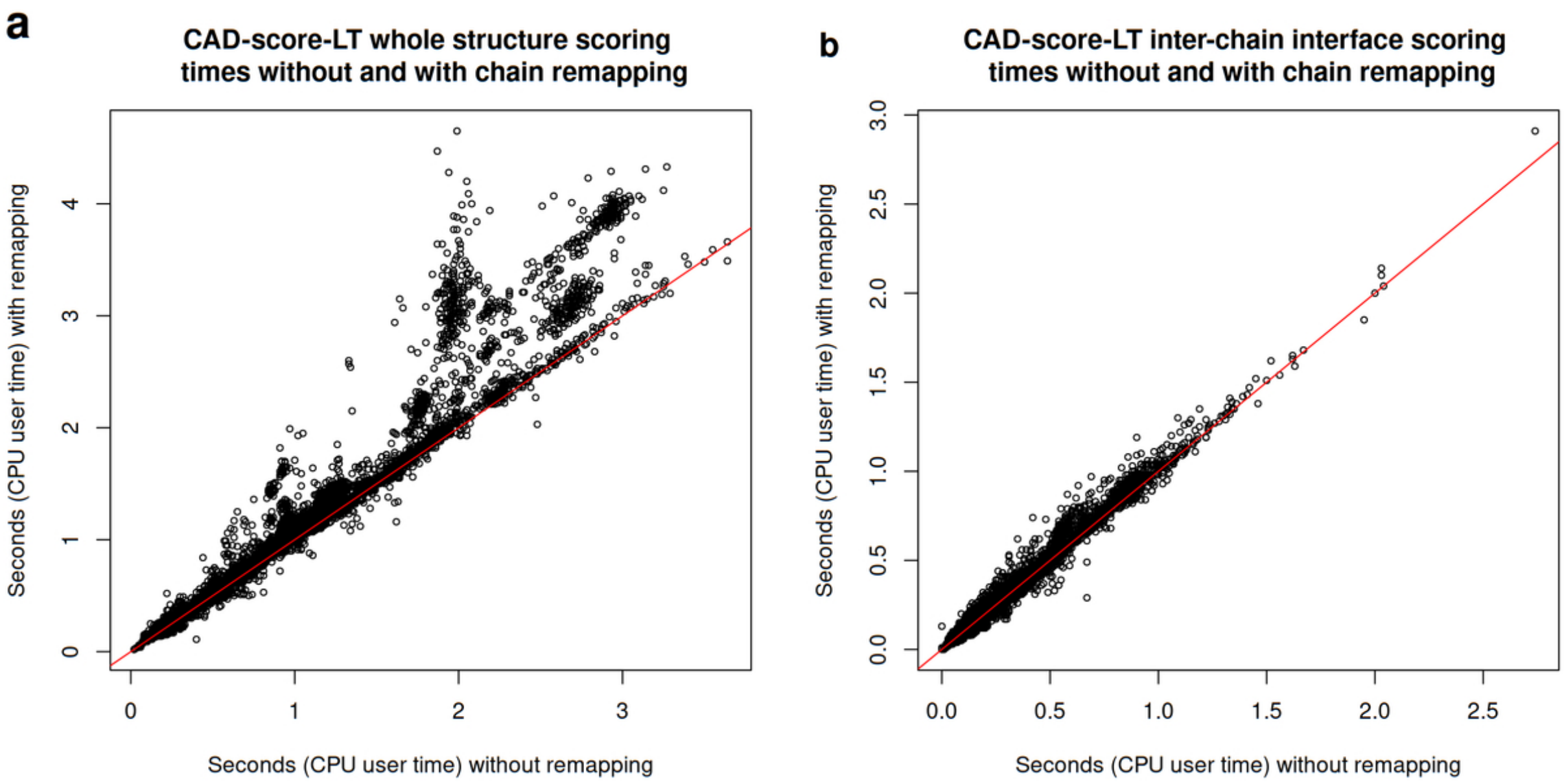

Fig. S6. Running times (CPU user times) of CAD-score-LT with and without automatic chain remapping, recorded for whole-structure (a) and inter-chain interface (b) pairwise comparisons.

S4 Benchmarking CAD-score-LT against other scores

Using the same input structural data as in sections S2 and S3, we used OpenStructure (https://openstructure.org) to calculate multiple pairwise scores commonly used to compare inter-chain interfaces:

- ilDDT (interface local Distance Difference Test);
- ICS (interface contacts similarity);
- IPS (interface patch similarity);
- QS-score (Quaternary Structure score) variants:
  - QS-global (considers the entire structure);
  - QS-best (considers the subset of residues and interfaces covered by both the model and reference);
- DockQ variants:
  - DockQ-wave (weighted average DockQ of all interfaces, not penalizing incomplete residue coverage in the model);
  - DockQ-wave-full (weighted average DockQ of all interfaces, penalizing incomplete residue coverage in the model);
- TM-score (Template Modeling score, based on structural superposition).

We ran OpenStructure in its default mode, which performs automatic chain remapping intended to identify chain matches that result in higher similarity scores. We compared these results with the CAD-score-LT inter-chain residue–residue scores, also calculated with automatic chain remapping, as described in section S3. Plots comparing the CAD-score values with the OpenStructure-calculated score values are presented in figures S7 and S8. The observed Pearson correlation coefficients between CAD-score and the OpenStructure-calculated scores are listed below in descending order:

- cor(CAD-score, ilDDT) = 0.98
- cor(CAD-score, ICS) = 0.95
- cor(CAD-score, QS-global) = 0.92
- cor(CAD-score, QS-best) = 0.90
- cor(CAD-score, DockQ-wave-full) = 0.89
- cor(CAD-score, IPS) = 0.86
- cor(CAD-score, DockQ-wave) = 0.85
- cor(CAD-score, TM-score) = 0.68

Because the inter-chain residue–residue CAD-score was most strongly correlated with ilDDT, we also analyzed how the two scores compare when calculated with and without automatic chain remapping. CAD-score-LT allows chain remapping to be enabled or disabled directly. OpenStructure did not provide a flag for disabling chain remapping, but it allowed explicit chain mappings to be supplied for each model, making it possible to preserve the original model chain names. We observed that when chain remapping was disabled for both scores (Fig. S9a), the correlation coefficient was the same as when chain remapping was enabled for both scores (Fig. S9d). When different chain-remapping regimes were used for the two scores (Fig. S9b and Fig. S9c), the correlation coefficient decreased from 0.98 to as low as 0.80. This indicates that, for this test case, the chain-remapping procedures used by CAD-score-LT and OpenStructure had similar effects on the resulting similarity scores, although this analysis does not establish that the selected chain mappings are identical or globally optimal.

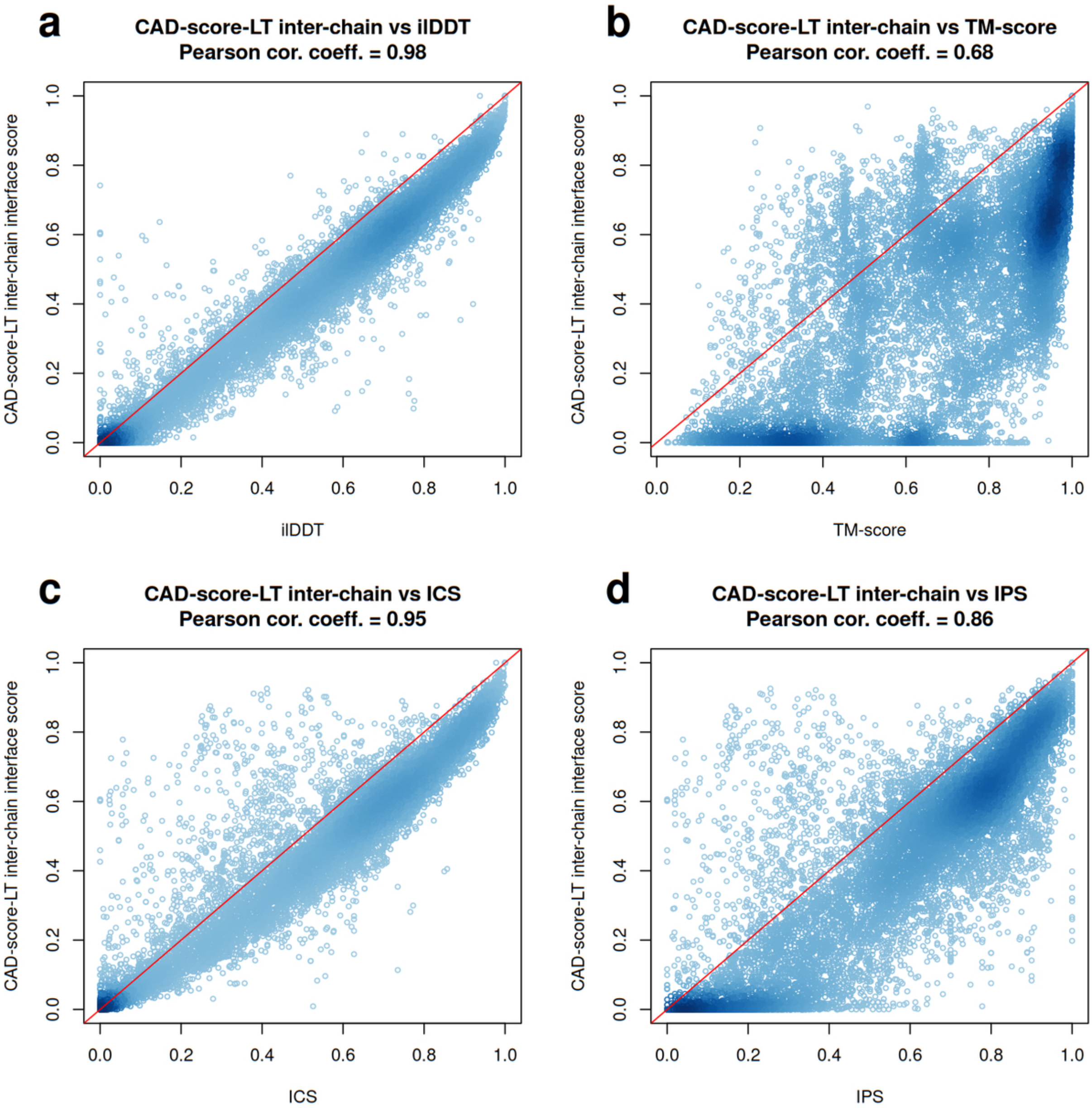

Fig. S7. Correlations between inter-chain interface scores produced by CAD-score-LT and OpenStructure for CASP16 oligomeric predictions: CAD-score vs ilDDT (a), CAD-score vs TM-score (b), CAD-score vs ICS (c), CAD-score vs IPS (d).

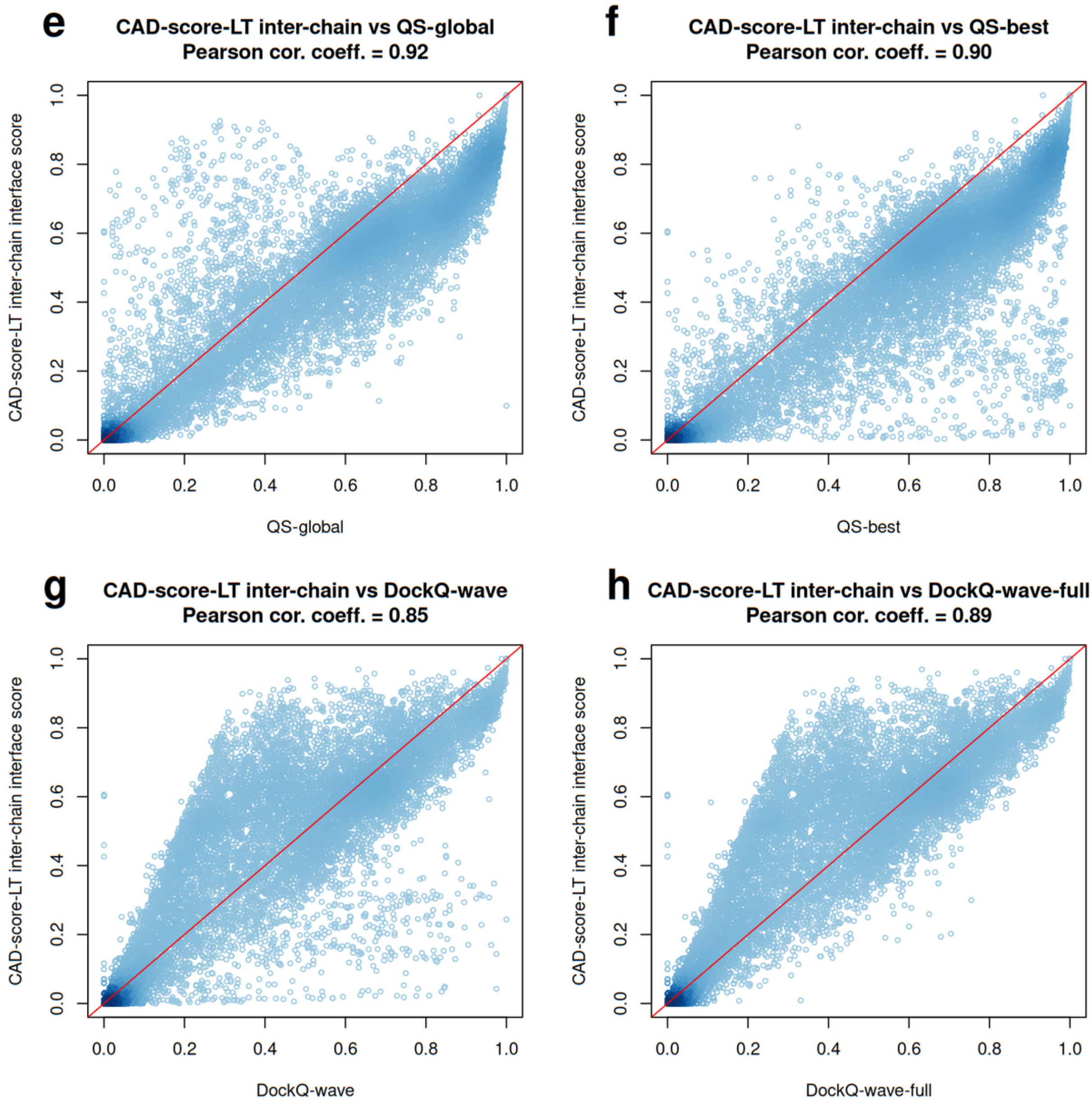

Fig. S8. Correlations between inter-chain interface scores produced by CAD-score-LT and OpenStructure for CASP16 oligomeric predictions: CAD-score vs QS-global (e), CAD-score vs QS-best (f), CAD-score vs DockQ-wave (g), CAD-score vs DockQ-wave-full (h).

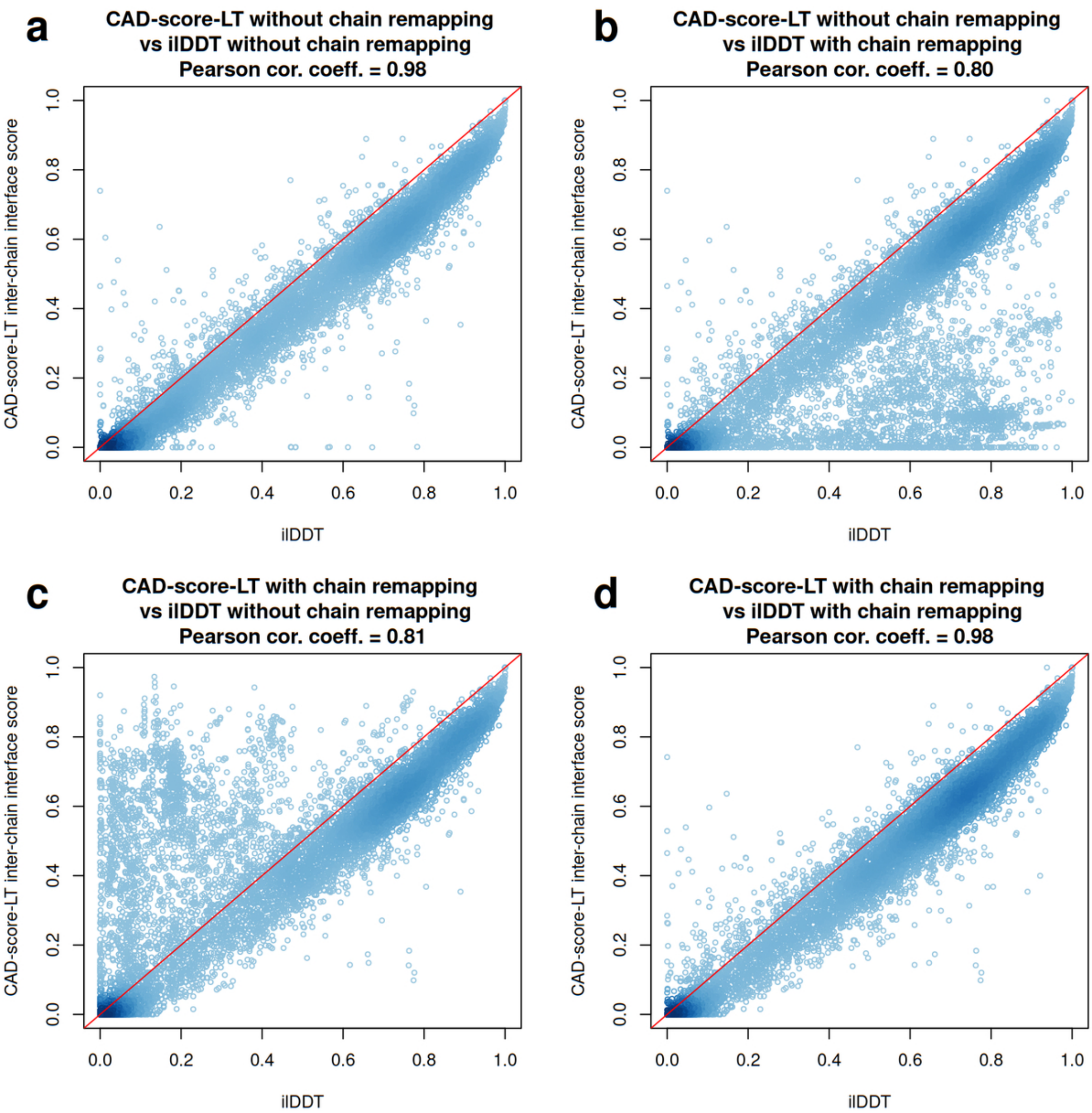

Fig. S9. Correlations between inter-chain interface scores produced by CAD-score-LT (with and without chain remapping) and ilDDT scores produced by OpenStructure (with and without chain remapping): CAD-score without chain remapping vs ilDDT without chain remapping (a); CAD-score without chain remapping vs ilDDT with chain remapping (b); CAD-score with chain remapping vs ilDDT without chain remapping (c); CAD-score with chain remapping vs ilDDT with chain remapping (d).

S5 Example application to a MassiveFold data set

To illustrate large-scale analysis, we applied CAD-score-LT to a MassiveFold model collection for CASP16 tetrameric target H1202 (stoichiometry A_2B_2), downloaded from https://entrepot.recherche.data.gouv.fr/api/access/datafile/608722. The set contained 8,040 structural models.

We performed all-against-all comparison of inter-chain interfaces using residue–residue contact CAD-scores in three modes:

1. direct comparison of raw input structures;
2. comparison after preprocessing with automatic residue renumbering and chain naming based on reference sequences and stoichiometry;
3. comparison after the same preprocessing followed by automatic chain remapping for each pairwise comparison.

Calculations were run on Ubuntu Linux 2024.04 on a 2018 workstation with Intel Core i9 processors, using eight processing threads. Running times (minutes:seconds) were 3:52 for mode 1, 4:16 for mode 2, and 22:22 for mode 3.

The score distributions are shown in Fig. S10. Preprocessing alone substantially improved the recovery of comparable interfaces, and additional chain remapping further shifted the distribution toward high-similarity pairs. The per-pair effect of chain remapping is shown in Fig. S11; many comparisons improved markedly after remapping.

We also clustered the models with the Taylor–Butina-like algorithm implemented in CAD-score-LT, using a CAD-score threshold of 0.5. Two dominant clusters were identified. The largest cluster contained 7,471 models and the second-largest cluster contained 251 models. Representative structures of these two clusters are shown in Fig. S12. Relative to the more frequent model class, the second class contains swapped regions in the larger chains, demonstrating that CAD-score-LT can rapidly separate major interface arrangements within very large model ensembles.

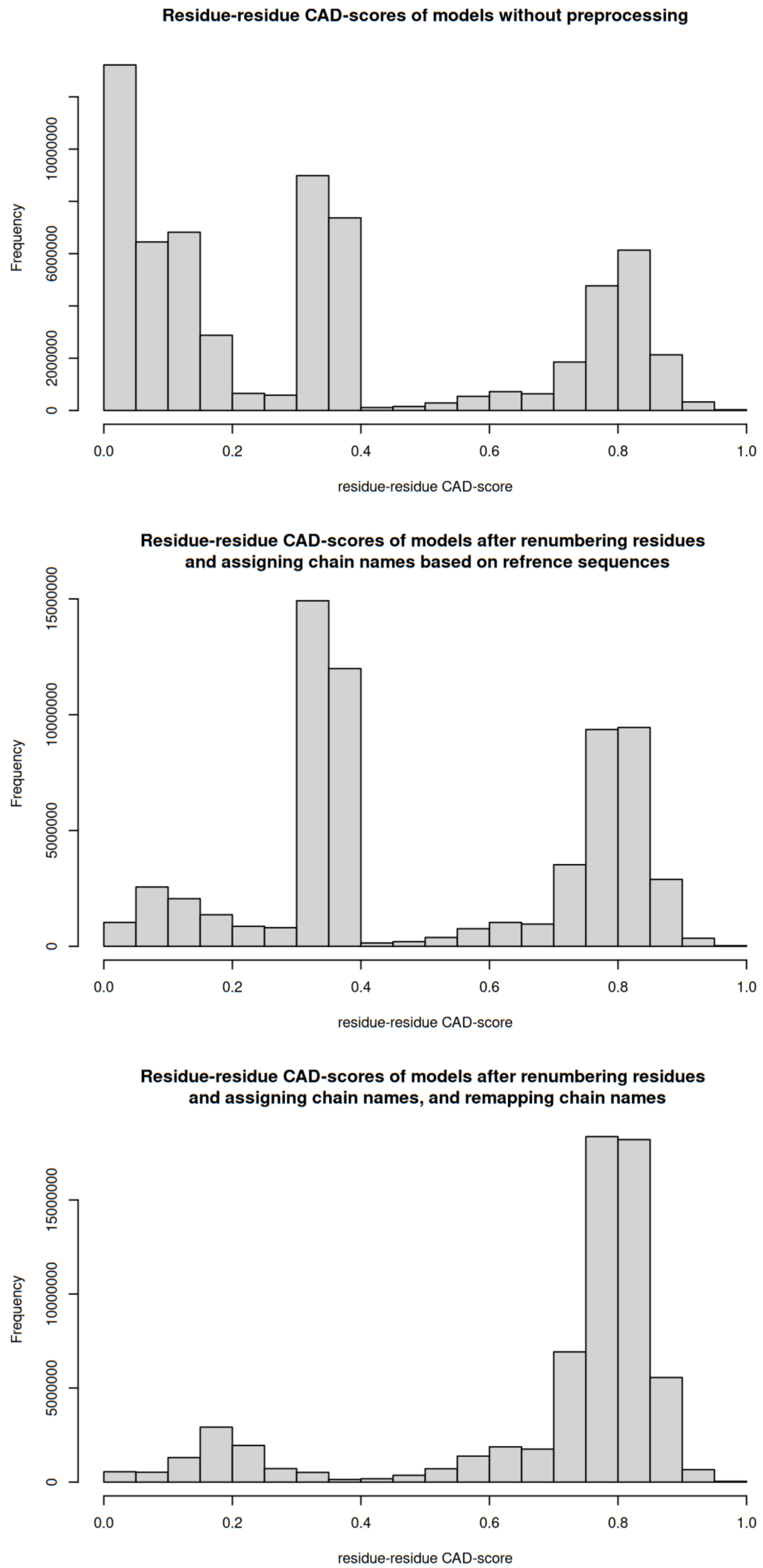

Fig. S10. Histograms of inter-chain interface CAD-scores based on residue–residue contacts from all-against-all comparison of 8,040 models in the MassiveFold set for CASP16 target H1202.

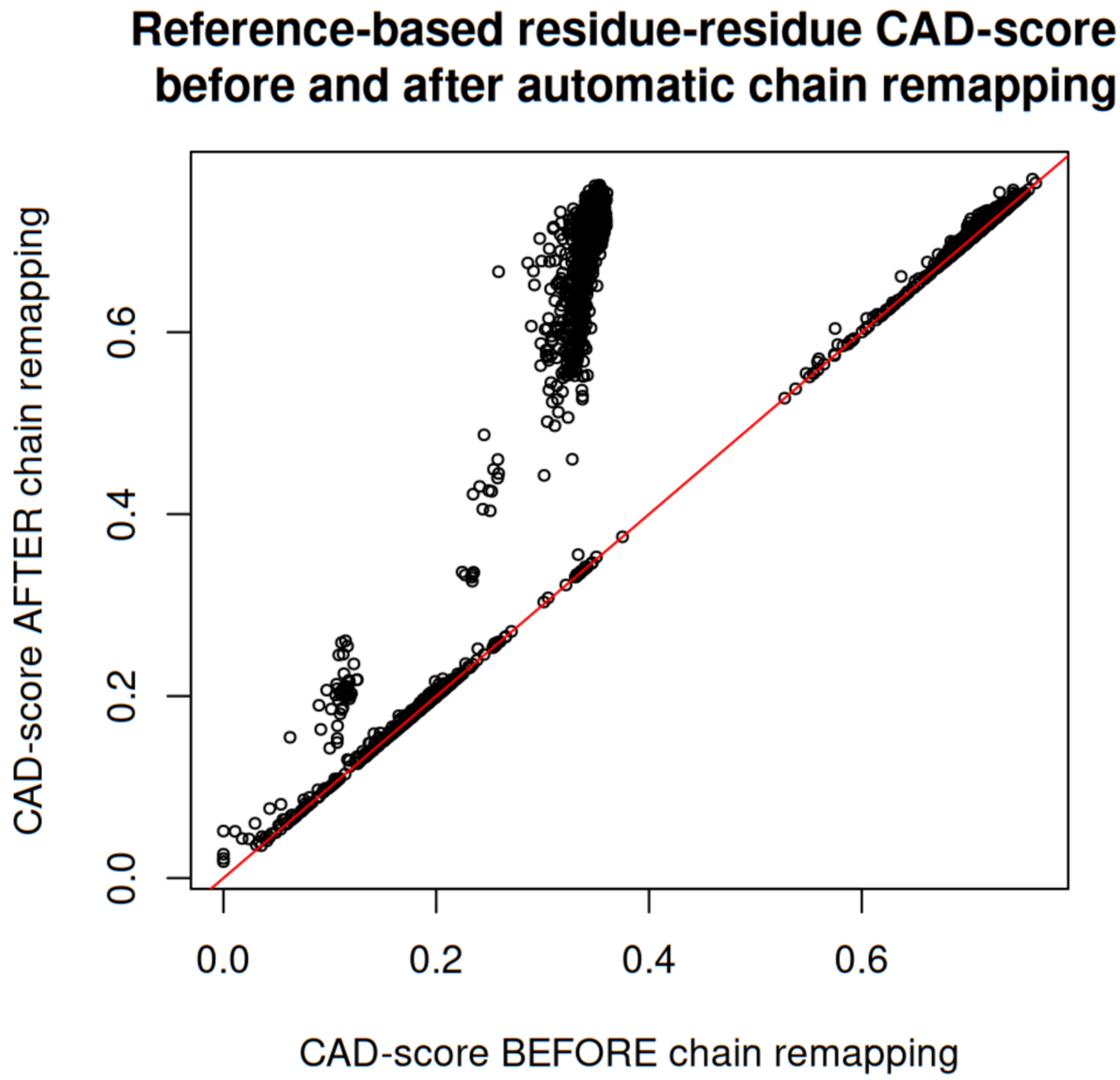

Fig. S11. Improvements of reference-based similarity scores due to automatic chain remapping in the MassiveFold set for CASP16 target H1202 (stoichiometry A_2B_2).

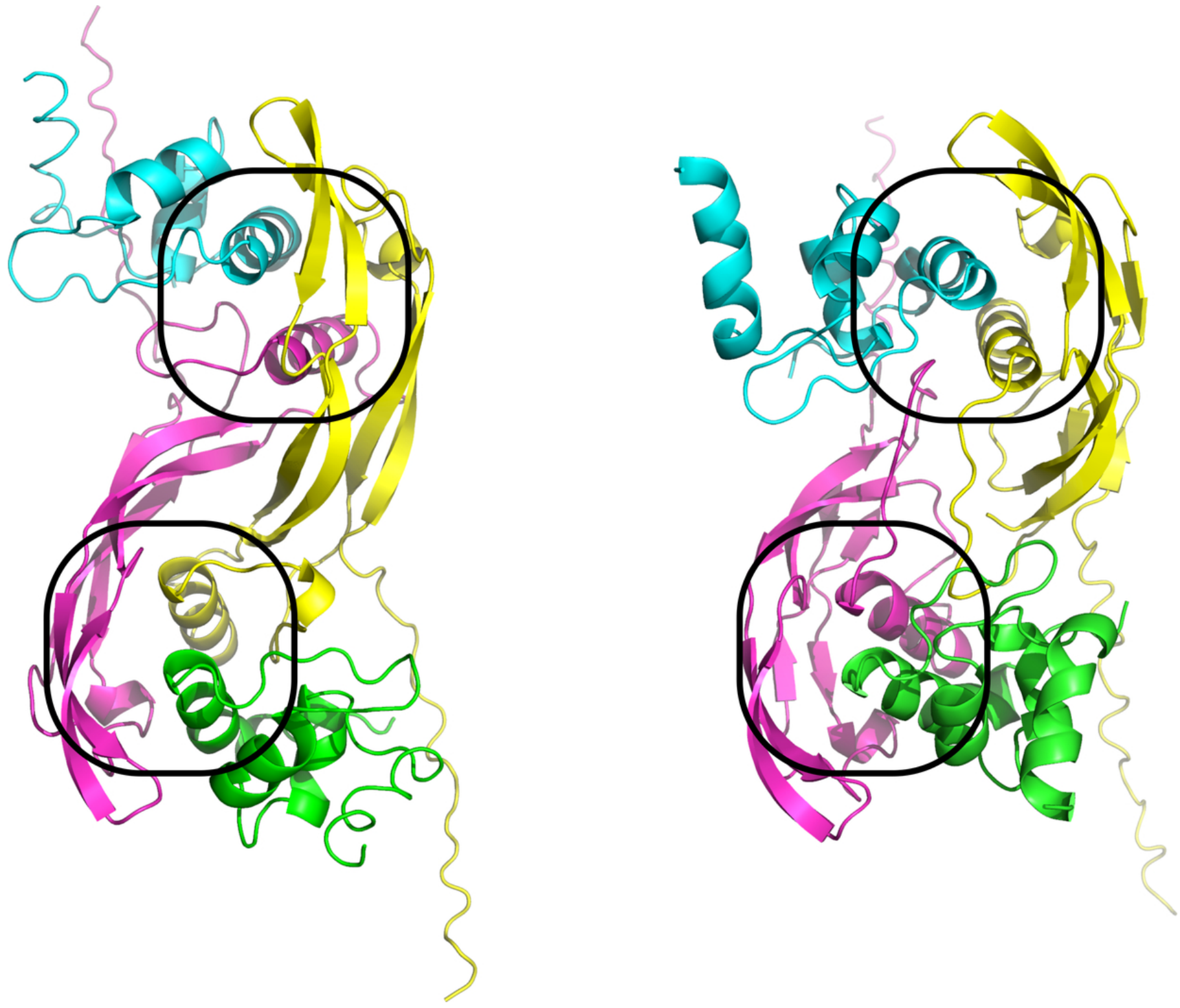

Fig. S12. Representative structures of the two dominant clusters in the MassiveFold set for CASP16 target H1202 (stoichiometry A_2B_2). Compared with the more frequent model on the left, the model on the right contains swapped regions in the larger chains (yellow and magenta).

S6 Example application to a set of large models with complex stoichiometry

In addition to the all-against-all analysis in section S5, we performed a similar analysis on a set of larger models with more complex stoichiometry.

We applied CAD-score-LT to a collection of predicted models for the CASP17 oligomeric target H2424 (stoichiometry $A_3B_3C_6D_{18}E_3F_3$), downloaded from https://predictioncenter.org/download_area/CASP17/predictions/oligo/H2424.tar.gz. The set contained 166 structural models, of which we considered the 122 models containing the complete set of chains. Each considered model contained more than 17,000 residues (more than 135,000 atoms).

We performed an all-against-all comparison of inter-chain interfaces using residue–residue contact CAD-scores in three modes:

1. direct comparison of the raw input structures;
2. comparison after preprocessing with automatic residue renumbering and chain naming based on reference sequences and stoichiometry;
3. comparison after the same preprocessing followed by automatic chain remapping for each pairwise comparison (also including clustering).

Calculations were run on Ubuntu Linux 2024.04 on a 2018 workstation with Intel Core i9 processors, using eight processing threads. Running times (hours:minutes:seconds) were 00:00:33 for mode 1, 00:00:39 for mode 2, and 1:34:33 (5673 seconds) for mode 3.

Mode 3 was substantially slower because every pairwise comparison included chain remapping for a complex stoichiometry. With 122 input models, $122 \times 121 = 14762$ pairwise comparisons were performed. On average, each pairwise comparison took $5673/14762 = 0.38$ seconds, which is practical for input complexes of this size. The running time can be further reduced by using more processors.

The score distributions for all three modes are shown in Fig. S13. Additional chain remapping was crucial for shifting the distribution toward high-similarity pairs.

The clustering analysis revealed that, at a CAD-score threshold of 0.5, there was a single dominant cluster containing 76 of the 122 models. Model H2424TS137_1 was its representative, defined as the model with the highest average similarity to the other cluster members. Identifying that main cluster would not be possible without the automatic chain remapping, because, as shown in Fig. S13, the overwhelming majority of pairwise comparisons would result in CAD-scores lower than 0.2.

Although the complete assembly in this example is heterooligomeric, it contains multiple copies of each chain type, including six copies of chain type C and 18 copies of chain type D. Thus, this example also tests chain remapping within high-order homooligomeric components, embedded in a larger heterooligomeric assembly.

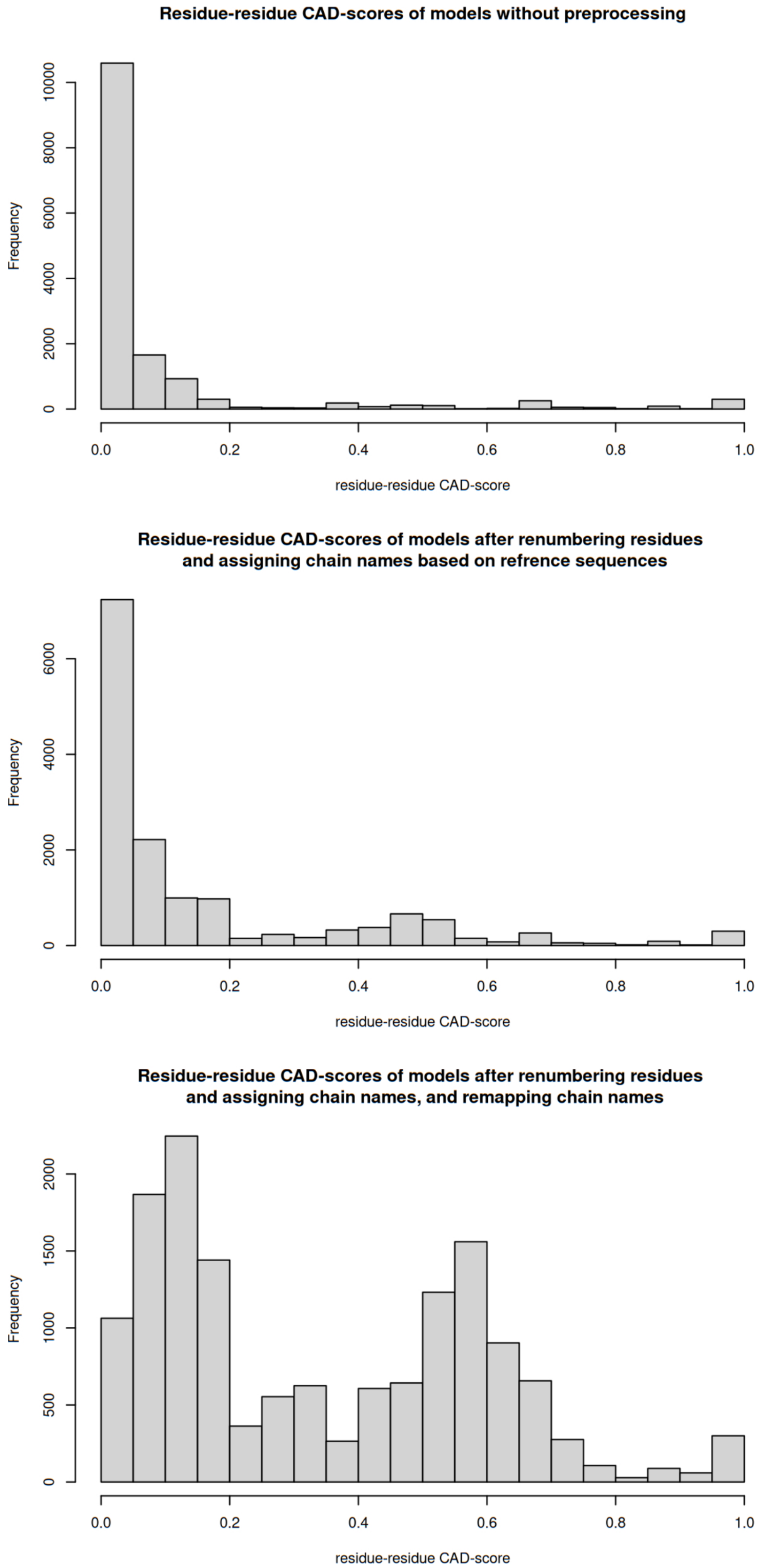

Fig. S13. Histograms of inter-chain interface CAD-scores based on residue–residue contacts from an all-against-all comparison of 122 large models with complex stoichiometry for the CASP17 target H2424.

S7 Example application to predictions of a protein–nucleic acid complex

We next applied CAD-score-LT to analysis of protein–protein and protein–DNA interfaces in CASP16 target M1239. The prediction models were downloaded from https://predictioncenter.org/download_area/CASP16/predictions/hybrid/M1239v1.tar.gz and https://predictioncenter.org/download_area/CASP16/predictions/hybrid/M1239v2.tar.gz. Models were compared with two target structures of identical sequence composition and stoichiometry ($A_4B_2C_2D_2E_2$), available in the PDB as 9DXD and 9DXF.

Inter-chain interface scoring was first performed with and without automatic chain remapping. Remapping often increased similarity scores substantially (Fig. S14), indicating that chain correspondence is a major confounder for this target and that automatic correction is necessary for meaningful reference-based assessment.

We then analyzed protein–protein and protein–DNA interfaces separately. Protein–protein interface quality was often poorly predictive of protein–DNA interface quality (Fig. S15a), whereas protein–DNA interface quality tracked more closely with overall interface quality (Fig. S15b). Thus, aggregate interface scores alone can mask distinct behavior of different interface classes.

Residue-level binding-site CAD-scores were also computed for both interface classes. Binding sites were generally reproduced better than detailed residue–residue contacts, both for protein–protein interfaces and for protein–DNA interfaces (Fig. S16). This pattern indicates that many models place the correct interacting regions in approximate contact even when fine contact geometry remains inaccurate.

We further computed chain–chain contact CAD-scores. As expected, agreement at chain level was usually higher than at residue-contact level because the chain-level descriptors summarize broader organization of an assembly (Fig. S17a–c). Interestingly, on the chain-level representation the protein–DNA interfaces were reproduced better than the protein–protein interfaces in most models (Fig. S17d).

The highest scores were obtained against target structure 9DXD. We therefore examined in detail model M1239v1TS110_1, which achieved the best protein–DNA interface quality. Residue–residue contact CAD-scores are summarized in Fig. S18. The protein–DNA interface scored higher than the protein–protein interface, although some DNA-end contacts were misplaced at the contact level. When the same model was evaluated on the binding site areas of the protein residues (Fig. S19), the DNA-interacting regions were recovered more accurately than suggested by contact-level scoring alone. Visual inspection indicates that some DNA segments are positioned with an approximate shift along the duplex, which preserves interacting regions better than precise residue–residue contacts.

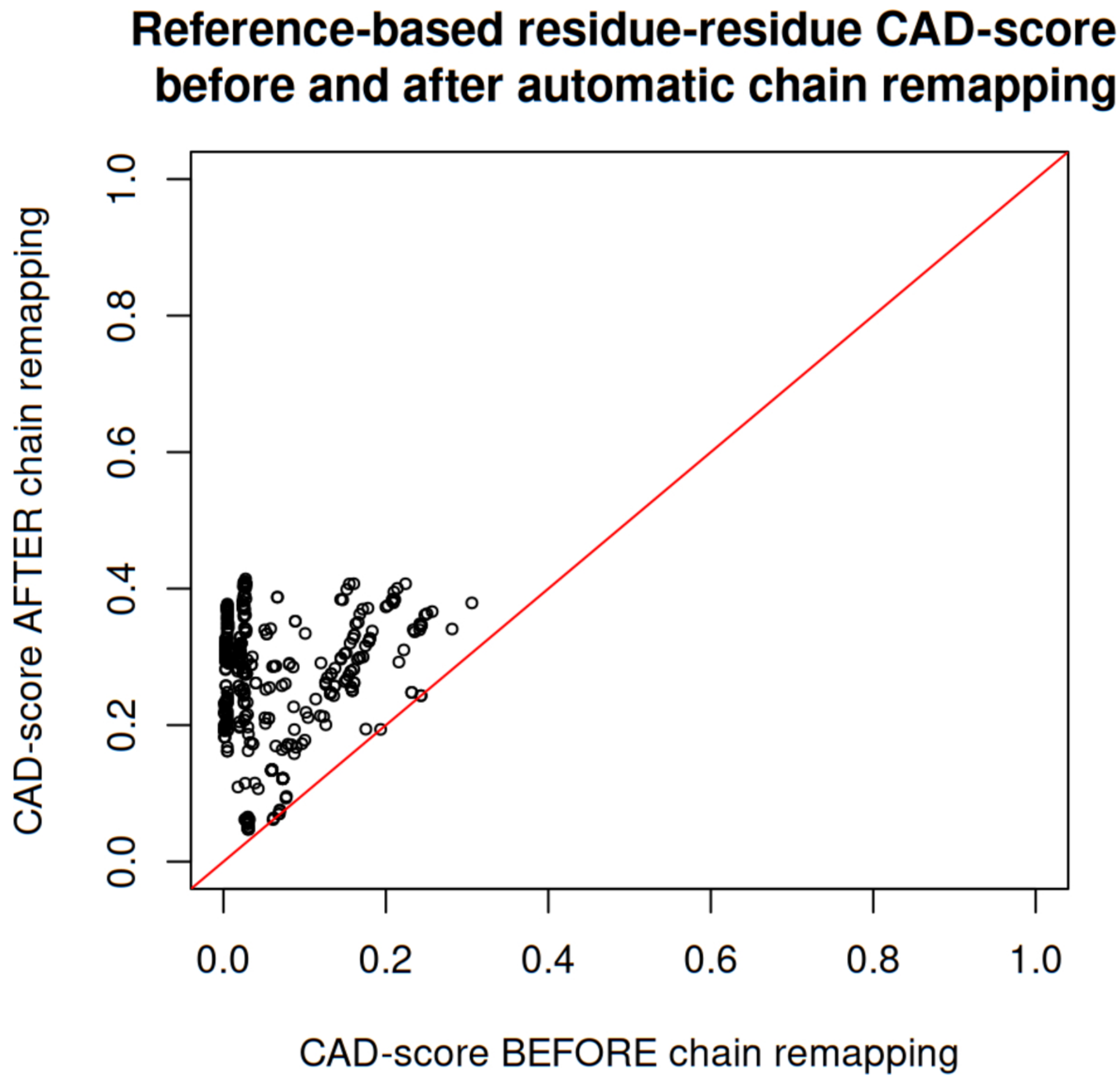

Fig. S14. Inter-chain interface similarity scores produced by CAD-score-LT for CASP16 target M1239 before and after automatic chain remapping.

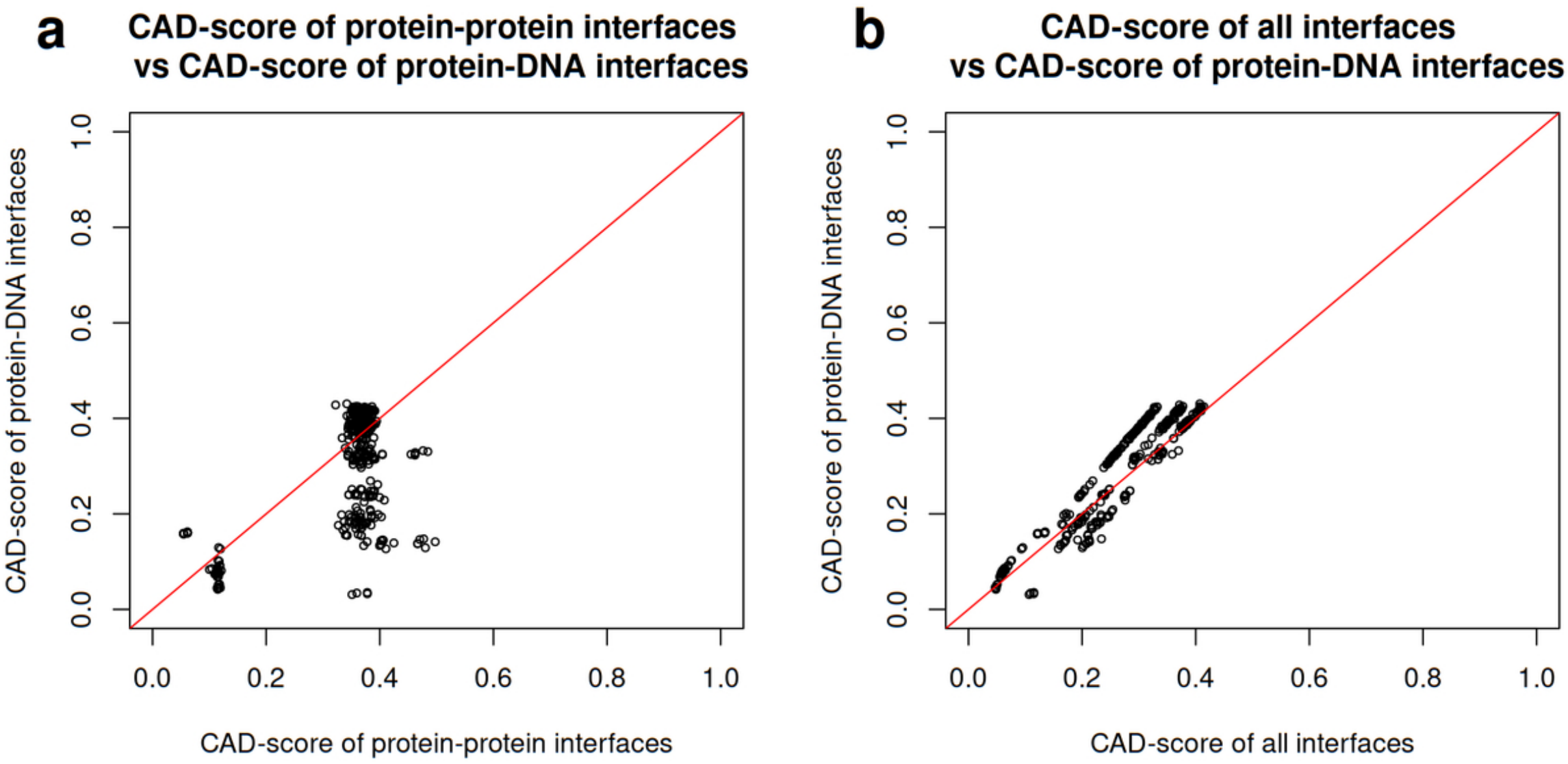

Fig. S15. Reference-based similarity scores produced by CAD-score-LT for different interface classes in models of CASP16 target M1239. Plots show the relationship between protein–protein and protein–DNA interface quality (a) and between overall interface quality and protein–DNA interface quality (b).

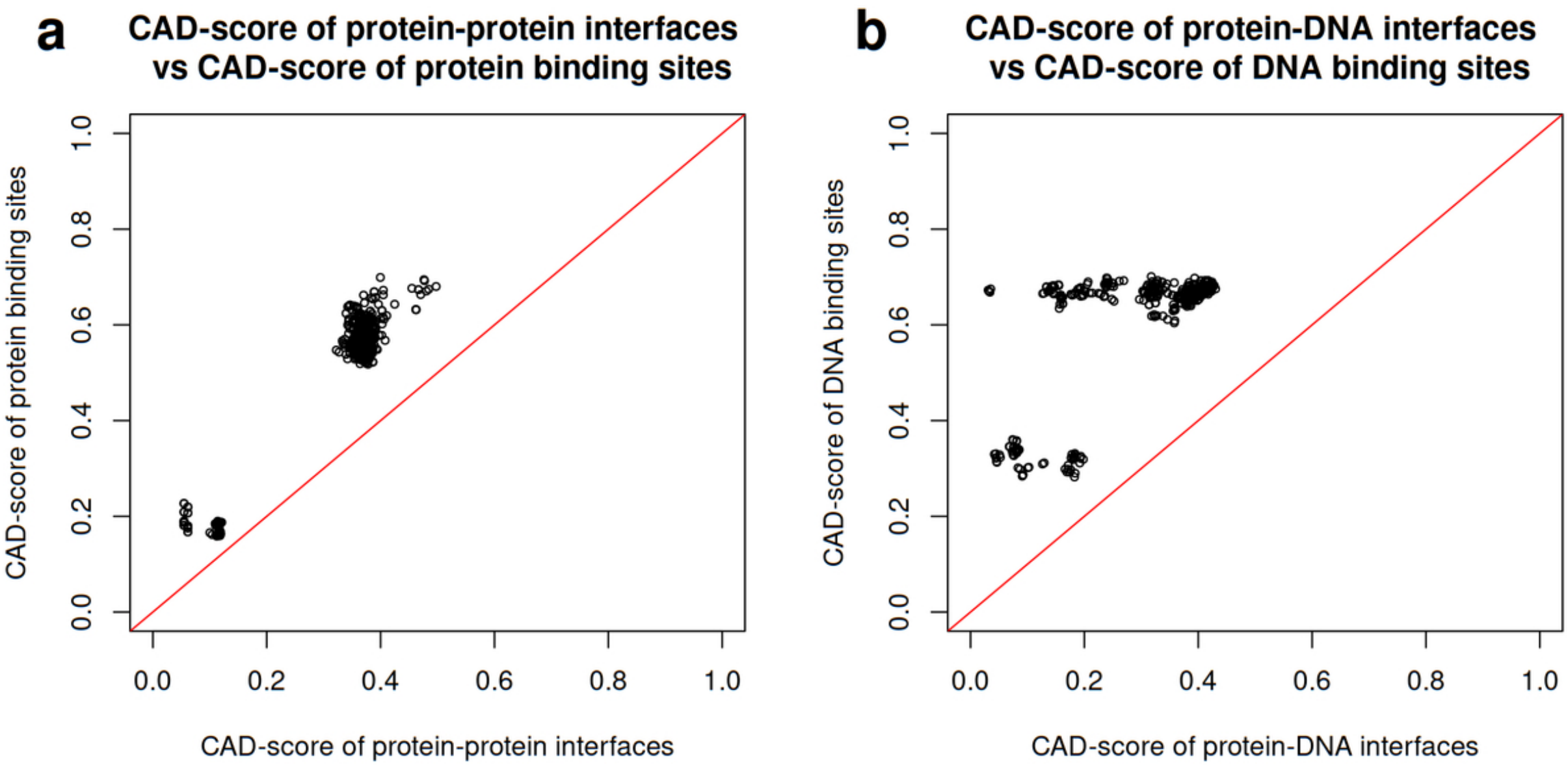

Fig. S16. Comparison of binding-site and interface quality values for models of CASP16 target M1239, shown for protein–protein interfaces (a) and protein–DNA interfaces (b).

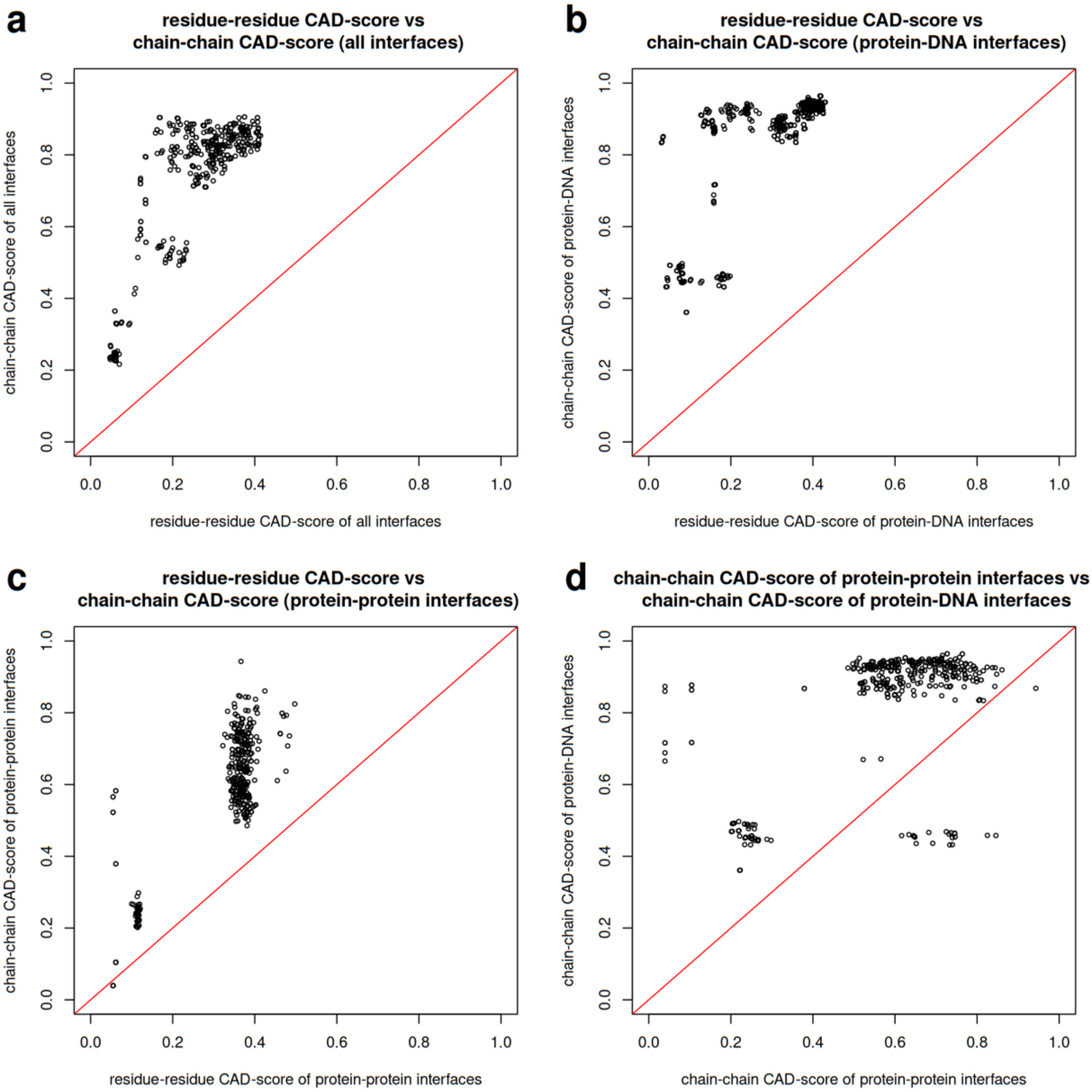

Fig. S17. Comparison of interface quality values on residue and chain levels for models of CASP16 target M1239.

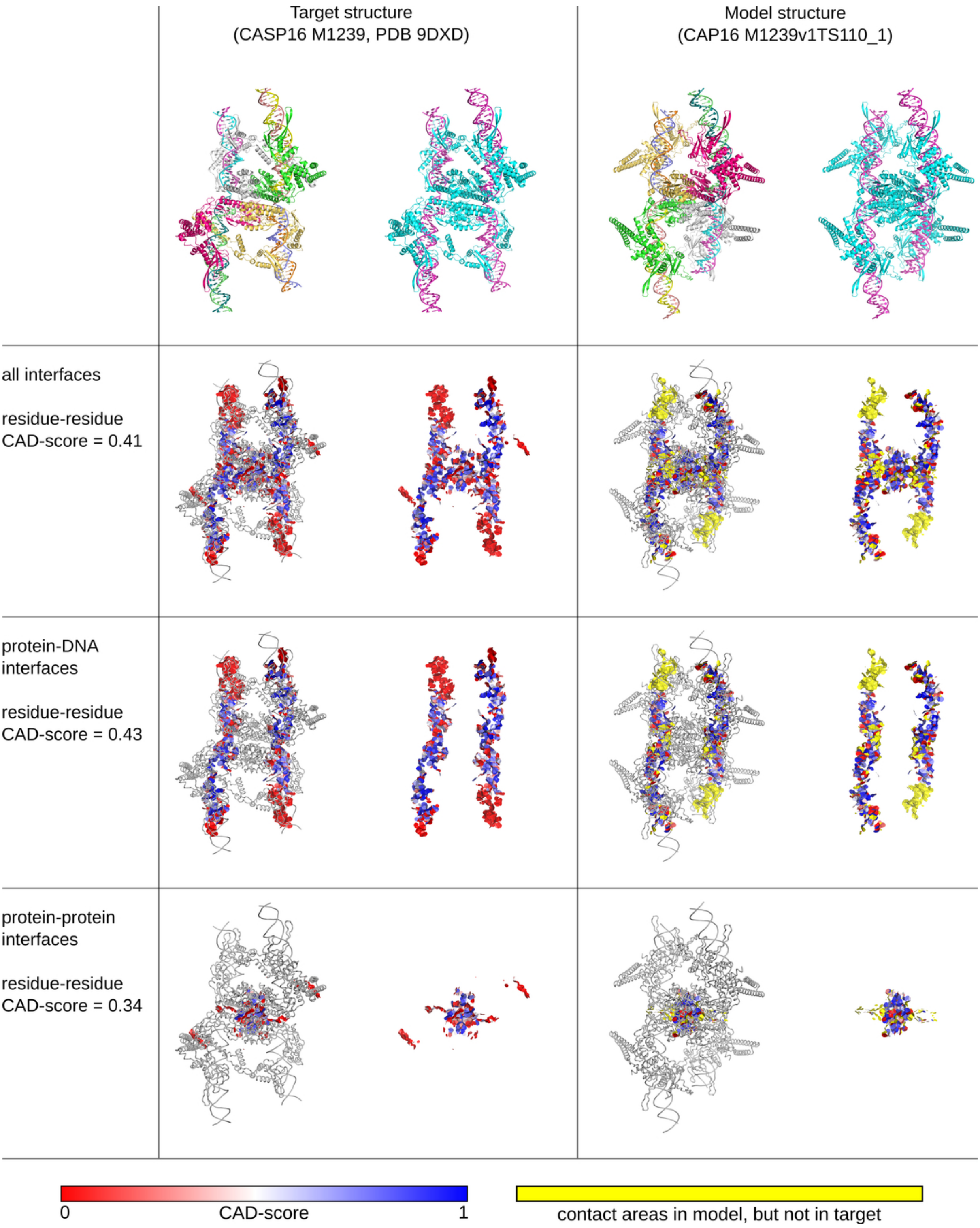

Fig. S18. Residue–residue contact CAD-score analysis of predicted interfaces in model M1239v1TS110_1, the model with the best protein–DNA interface quality for CASP16 target M1239. Protein–protein and protein–DNA contacts are colored by local residue–residue CAD-score values and displayed on both target and model structures.

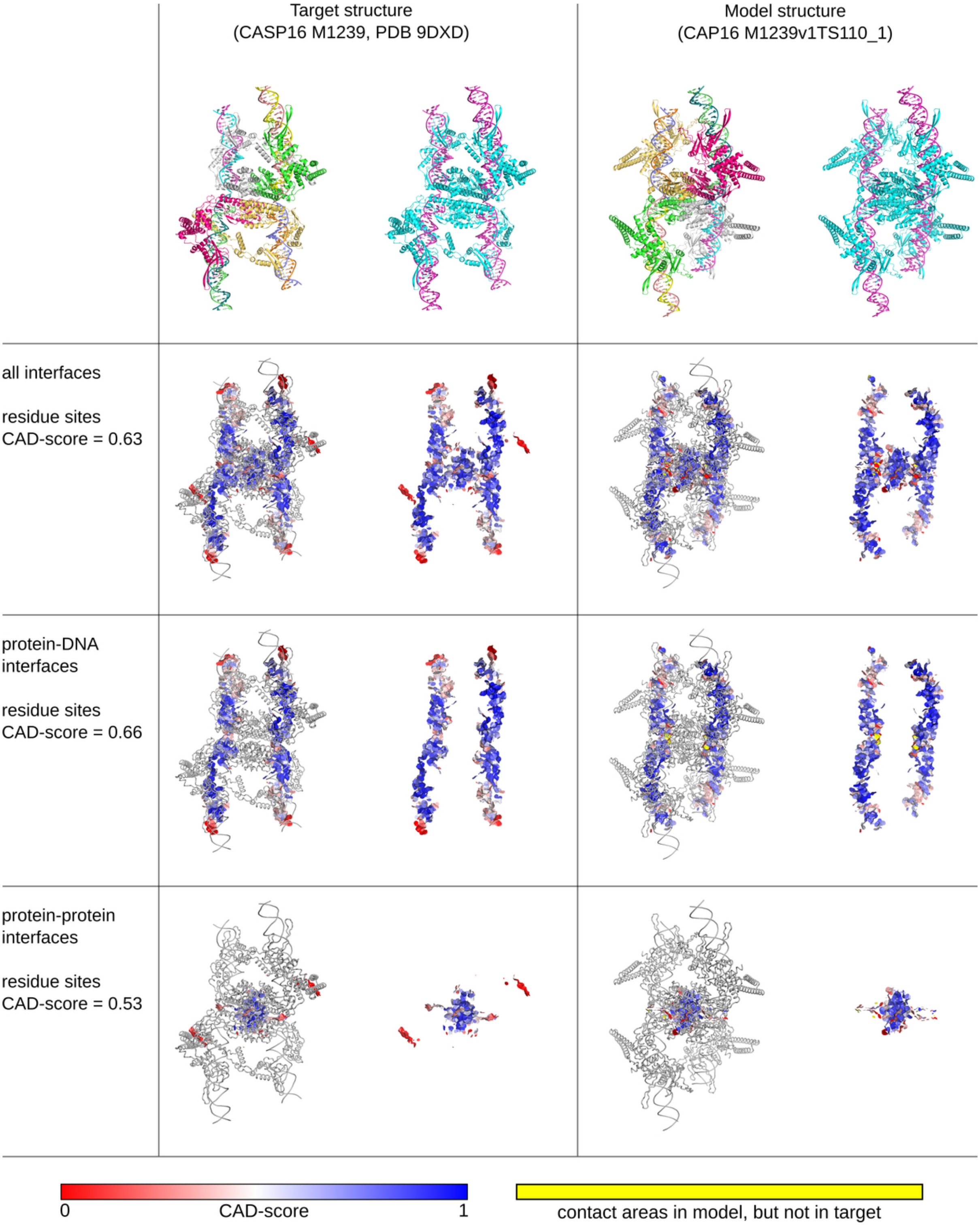

Fig. S19. Protein-side residue-level binding-site CAD-score analysis of predicted interfaces in model M1239v1TS110_1, the model with the best protein–DNA interface quality for CASP16 target M1239. Protein–protein and protein–DNA interfaces are colored by local protein-side residue-level binding-site CAD-score values and displayed on both target and model structures.

S8 Example application to protein–small molecule ligand docking models

Finally, we applied CAD-score-LT to protein–small molecule docking decoys for 284 targets from the CASF-2016 data set. RDKit-based companion scripts were used to prepare consistent ligand atom names and to derive ligand atom equivalence classes for symmetry-adjusted scoring. We then calculated protein–ligand interface atom–atom contact CAD-scores and atom-level binding-site CAD-scores, both with and without symmetry adjustment.

Enabling symmetry adjustment often increased similarity values substantially (Fig. S20), showing that chemically equivalent ligand atoms can strongly affect direct atom-level comparison if not treated explicitly.

We also compared CAD-score values with symmetry-adjusted RMSD values from CASF-2016. Atom–atom contact CAD-scores (Fig. S21a) were more stringent and provided finer resolution among low-RMSD decoys, whereas atom binding-site CAD-scores (Fig. S21b) were generally high once low-RMSD poses recovered the correct ligand-contacting region.

Despite these distinct behaviors, the mean per-target Spearman correlation with symmetry-adjusted RMSD was similar for the two CAD-score variants: -0.868 for atom–atom contact CAD-score and -0.865 for atom binding-site CAD-score. Histograms of per-target Spearman coefficients are shown in Figs. S22 and S23.

Figure S24 illustrates a representative disagreement between atom–atom CAD-score and symmetry-adjusted RMSD. The magenta-colored decoy has better RMSD but worse CAD-score than the cyan-colored decoy. Visual inspection shows that the cyan-colored decoy reproduces ligand ring placement and the corresponding protein–ligand contact pattern more accurately, whereas the magenta-colored decoy gains in RMSD mainly because its flexible tail more closely matches the target. This example highlights the complementary information provided by contact-area-based scoring.

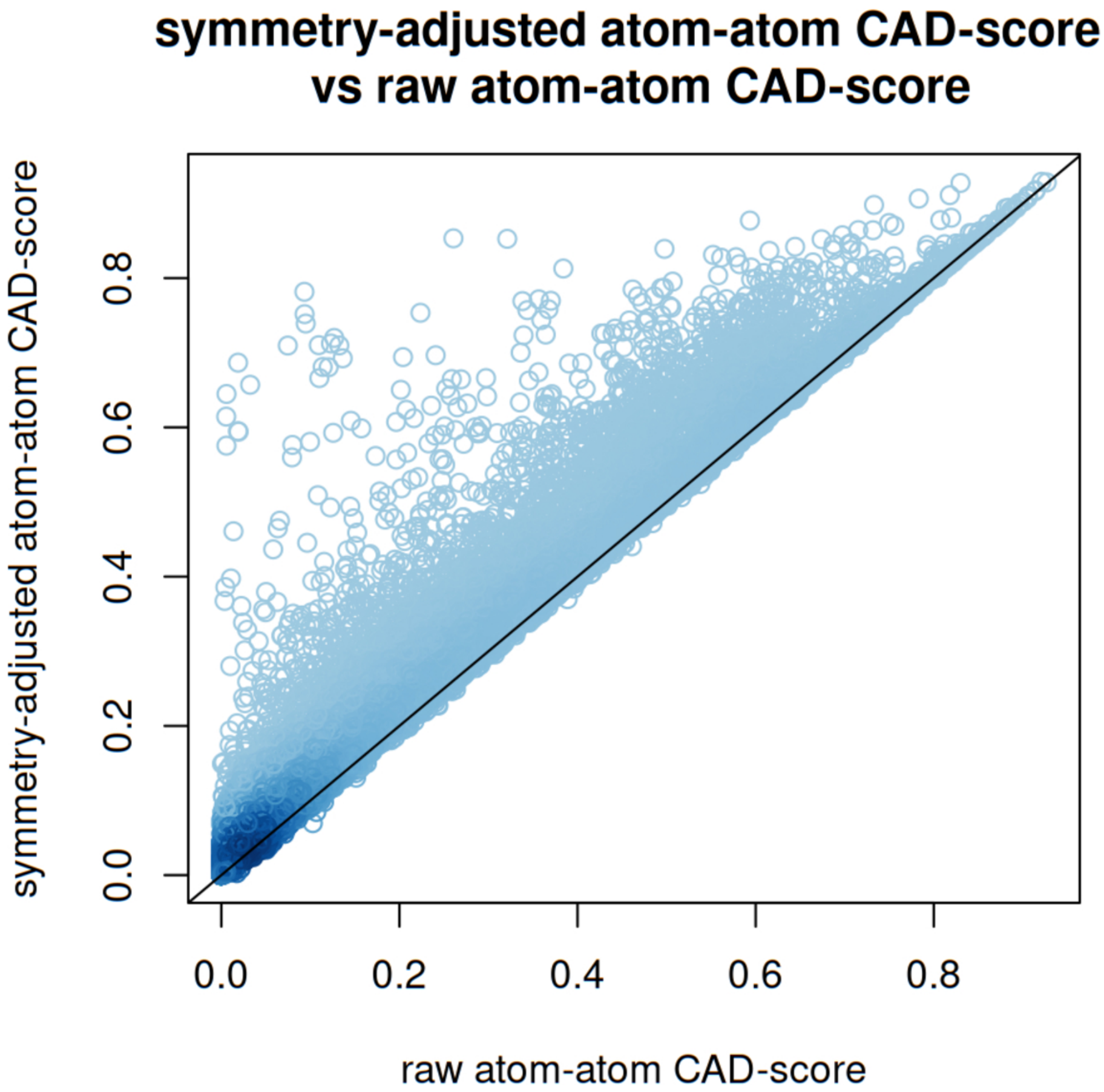

Fig. S20. Comparison of raw and symmetry-adjusted CAD-score values for protein–ligand decoys from the 284 targets in the CASF-2016 data set.

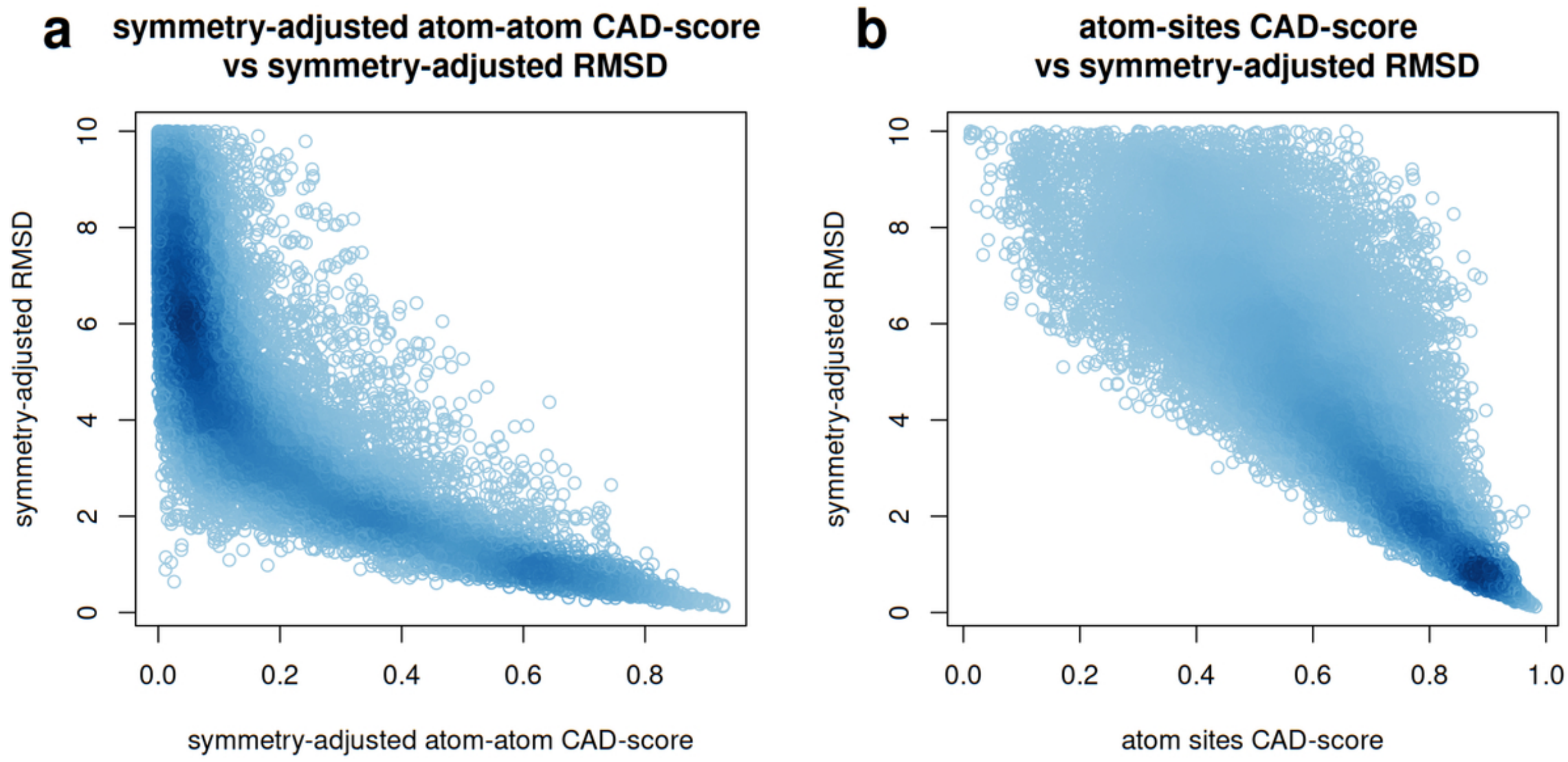

Fig. S21. Comparison of symmetry-adjusted protein–ligand CAD-score values with symmetry-adjusted RMSD values from CASF-2016, shown for atom–atom contact CAD-score (a) and atom binding-site CAD-score (b).

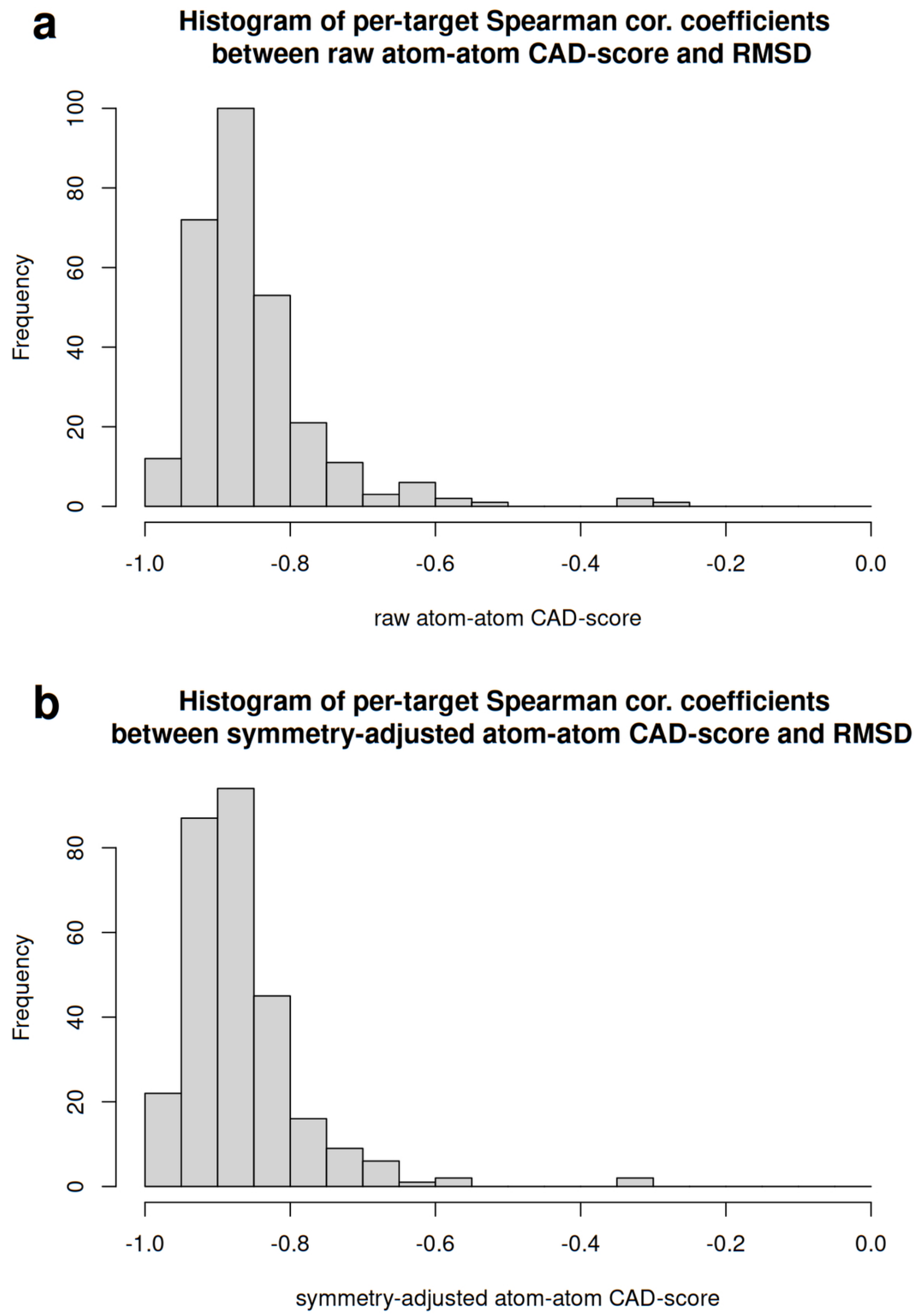

Fig. S22. Histograms of per-target Spearman correlation coefficients for atom–atom contact CAD-score without (a) and with (b) atom symmetry adjustment.

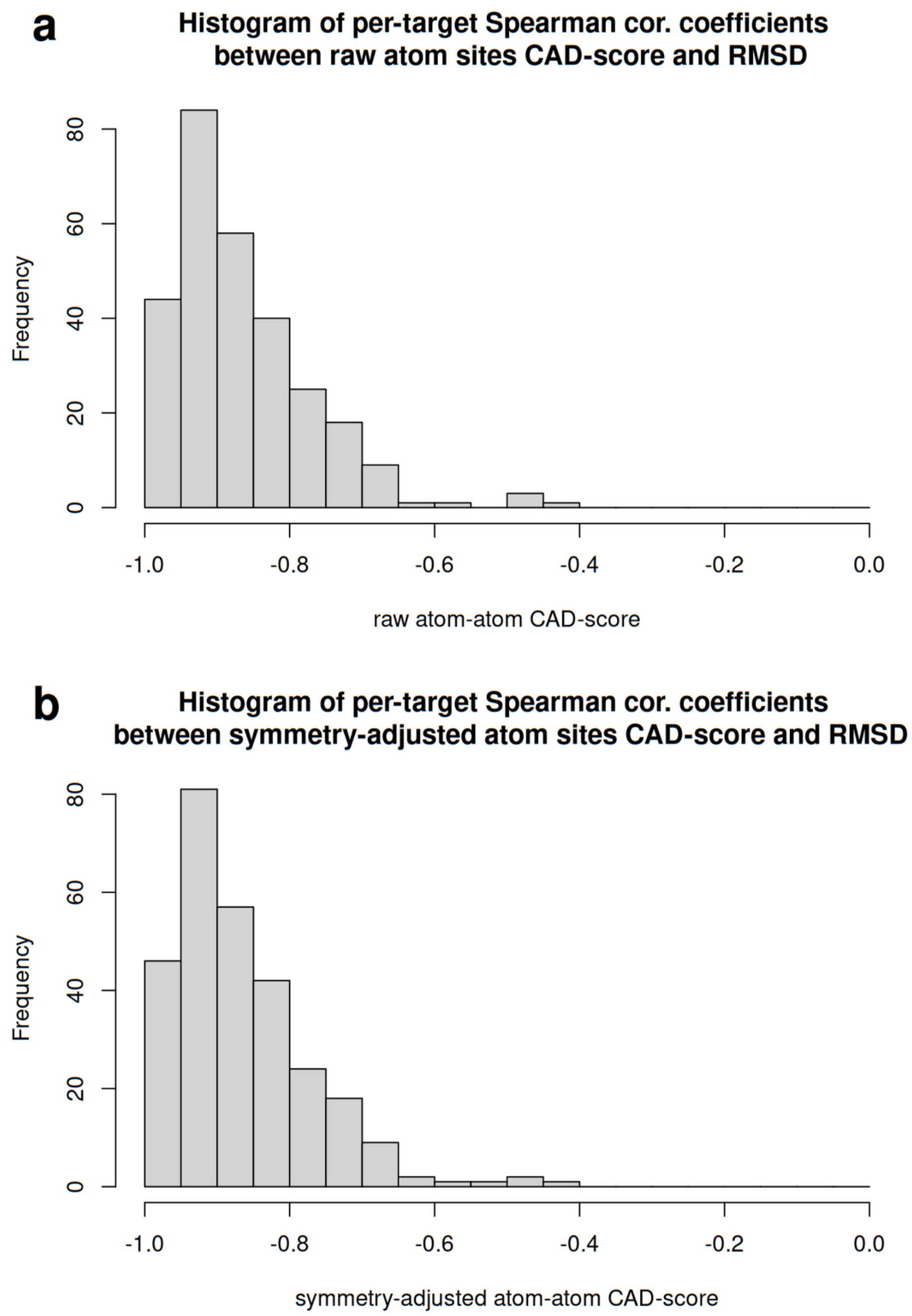

Fig. S23. Histograms of per-target Spearman correlation coefficients for atom binding-site CAD-score without (a) and with (b) atom symmetry adjustment.

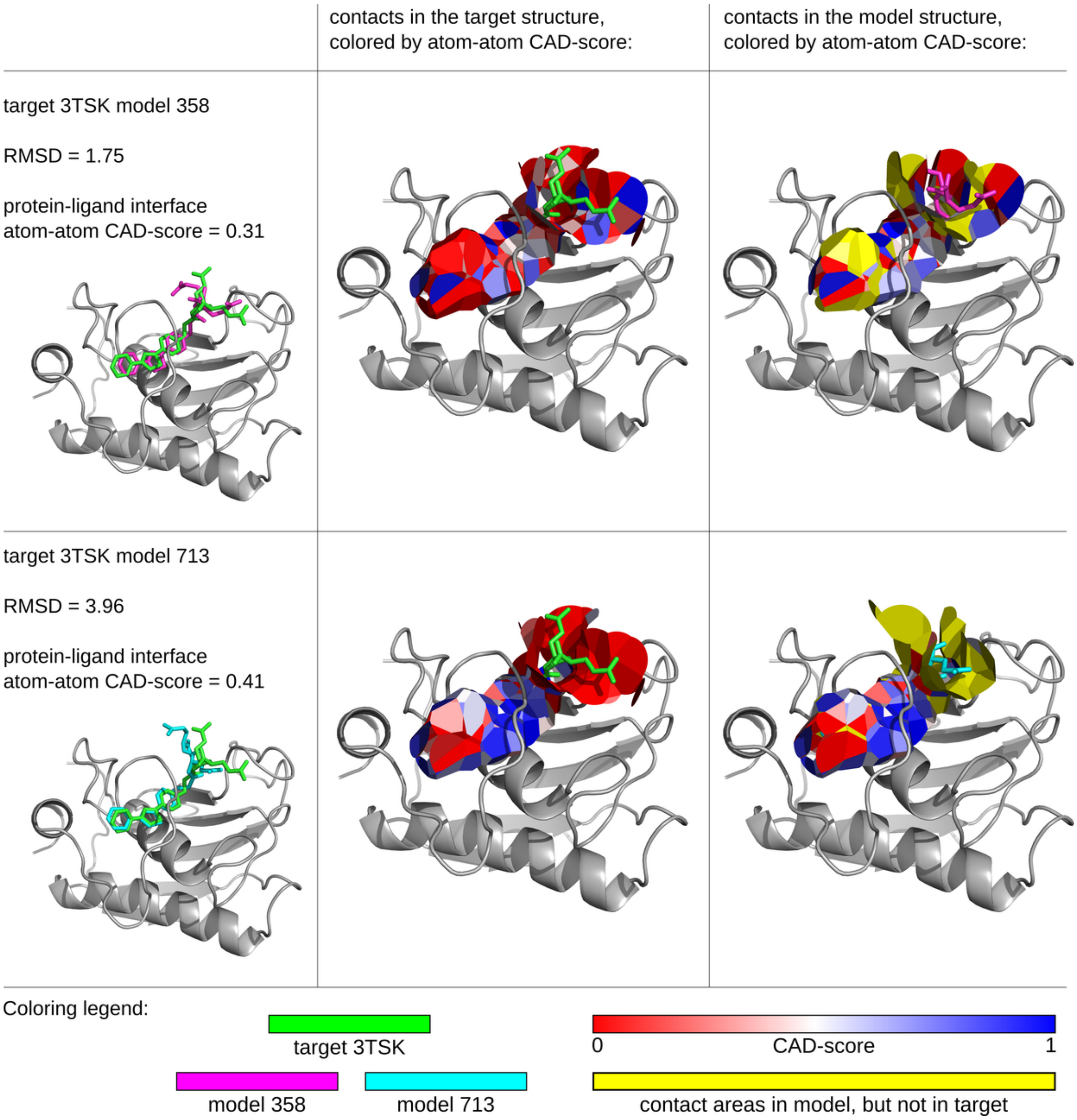

Fig. S24. Analysis of atom–atom contact CAD-score values for two CASF-2016 docking decoys of the human MMP12 complex with an L-glutamate-motif inhibitor from PDB entry 3TSK. Protein–ligand contacts are colored by local atom–atom CAD-score values and displayed on both target and model structures.
